# Regulation of Citron kinase by CDK1 and Aurora B regulates midbody formation and stability

**DOI:** 10.1242/jcs.264556

**Published:** 2026-05-22

**Authors:** Luisa Capalbo, Ella F. J. Halcrow, Zuni I. Bassi, Pier Paolo D'Avino

**Affiliations:** Department of Pathology, University of Cambridge, Tennis Court Road, Cambridge, CB2 1QP, UK

**Keywords:** Abscission, Cell division, Cytokinesis, Midbody, Phosphorylation

## Abstract

Many cell division events are regulated by protein phosphorylation, which can result from cross-regulatory mechanisms among mitotic kinases and phosphatases that have yet to be fully elucidated. Here, we report the characterization of a novel mechanism by which CDK1 and Aurora B (AURKB) kinases regulate the distribution and interactions of Citron kinase (CIT-K, encoded by *CIT*). We show that CDK1 phosphorylates serine 440 and AURKB phosphorylates serine 699, both residues located adjacent to or within the CIT-K coiled coil domain. S440 and S699 temporal phosphorylation profiles reflect the activity of the kinases responsible for their phosphorylation. Functional analyses using phosphorylation site mutants indicate that S699 phosphorylation is important for CIT-K localization and successful cytokinesis, whereas perturbing S440 phosphorylation leads to abnormal midbody formation and accumulation of post-mitotic midbody remnants (MBRs). Furthermore, we found that phosphorylation at either residue reduces the ability of CIT-K to interact with its midbody partners AURKB, KIF14 and MKLP1 (also known as KIF23). Taken together, our findings indicate that phosphorylation of CIT-K by CDK1 and AURKB regulates midbody formation and MBR stability by controlling the association of CIT-K with its partners. They expand our understanding of the mechanisms that regulate abscission and can lead to further insights into the role of MBRs in post-mitotic events.

## Teaser

**Summary:** Phosphorylation of Citron kinase by Aurora B and CDK1 regulates midbody formation and stability by controlling the association of Citron kinase with its partners.

## INTRODUCTION

Cell division is responsible for the correct partitioning of genomic and cytoplasmic contents between the two nascent daughter cells. The entire mitotic process is regulated by finely tuned signals that control the activity, localization, function and interactions of proteins and protein complexes (Wieser and Pines, 2015). Due to the rapidity of mitotic events and chromatin inaccessibility, most mitotic processes are regulated by reversible post-translational modifications, such as phosphorylation. The changes in the phosphorylation status of mitotic proteins are controlled by opposing enzymes, kinases and phosphatases, and these regulate every mitotic event, including entry into mitosis, the assembly of the mitotic spindle, chromosome alignment and segregation, and daughter cell separation during cytokinesis (Cuijpers and Vertegaal, 2018; Gelens et al., 2018; Holder et al., 2019; Nasa and Kettenbach, 2018).

The last phase of cell division, cytokinesis, represents a powerful temporal window to study phosphorylation for various reasons. Anaphase and cytokinesis are triggered by the degradation of cyclin B (CCNB1) and consequent inactivation of its partner, cyclin-dependent kinase 1 (CDK1). This drop in CDK1 activity is complemented by increased activity of PP1 and PP2A serine/threonine phosphatases as well as by changes in the localization of other mitotic kinases, including Aurora B (AURKB), Citron kinase (CIT-K, encoded by *CIT*) and Polo-like kinase 1 (Plk1) (D'Avino and Capalbo, 2016; Holder et al., 2019). Together, these events lead to changes in the phosphorylation and activity of many proteins that drive a complete re-organization of the cytoskeleton (D'Avino et al., 2015). First, cells establish the position of the division plane through signals emanating from the spindle microtubules (MTs), which are then re-organized into an array of antiparallel and interdigitating MTs, known as the central spindle, during anaphase. Spindle MTs also promote furrow ingression through the assembly and constriction of an actomyosin contractile ring, which compacts the central spindle and leave the daughter cells connected by an intercellular bridge in late cytokinesis. An electron- and phase-dense structure, first described by Flemming more than a century ago (Flemming, 1891), forms at the center of the intercellular bridge (Guizetti and Gerlich, 2010). This organelle, the midbody, is composed of an assortment of proteins with diverse functions that are either former components of the contractile ring and central spindle, or specifically recruited during the midbody maturation process that ultimately leads to the final physical separation or abscission of the two daughter cells (D'Avino and Capalbo, 2016; Mierzwa and Gerlich, 2014). Midbody proteins are arranged in a very precise and stereotyped spatial pattern along the midbody (Hu et al., 2012), which can be divided in approximately three major regions: the midbody ring, the midbody central core and the midbody arms, which flank the midbody core (D'Avino and Capalbo, 2016). The proper localization, regulation and interactions of midbody proteins are essential for the execution of abscission and for preventing incorrect genome segregation (Mierzwa and Gerlich, 2014). The large multifunctional kinase CIT-K plays a key evolutionarily conserved role in cytokinesis by maintaining the orderly arrangement of midbody proteins (Bassi et al., 2013; D'Avino, 2017; McKenzie et al., 2016). CIT-K exerts this role by interacting and regulating the localization of several midbody proteins, including anillin, the centralspindlin complex [a heterotetramer composed of two subunits of the kinesin MKLP1 (also known as KIF23) and two molecules of RacGAP1], the chromosomal passenger complex (CPC, of which AURKB is the kinase subunit), the kinesins KIF14 and KIF20A, and the GTPase RhoA (Bassi et al., 2013, 2011; Capalbo et al., 2019; Gai et al., 2011; Gruneberg et al., 2006; McKenzie et al., 2016). CIT-K has been shown to be important for brain development and spermatogenesis in rodents and variants in the *CIT* gene cause primary microcephaly in humans (Di Cunto et al., 2000, 2002; Harding et al., 2016; Li et al., 2016; Moawia et al., 2017; Sarkisian et al., 2002; Shaheen et al., 2016). Furthermore, CIT-K has been implicated in DNA damage repair and carcinogenesis (Iegiani et al., 2025; Meng et al., 2019; Pallavicini et al., 2020; Rawat et al., 2023).

Recent studies have revealed that the midbody also has important functions post mitosis. After abscission, the midbody remnants (MBRs) can be either reabsorbed by one of the daughter cells or released into the extracellular environment and then eventually internalized by another cell (Crowell et al., 2014; Peterman et al., 2019). These post-mitotic MBRs have been implicated in diverse biological processes, including cell fate, pluripotency, apical–basal polarity, tissue organization, cell proliferation, and cilium and lumen formation (Dionne et al., 2015; Peterman and Prekeris, 2019). Recent studies have indicated that MBRs are large extracellular vesicles that contain ribonucleoprotein complexes characterized by distinct populations of mRNAs and spatiotemporally regulated translation (Park et al., 2023; Rai et al., 2021; Suwakulsiri et al., 2024). Therefore, MBRs can function as intercellular signaling organelles that can influence the function, activity and properties of neighboring cells (Kuriyama et al., 2025). In support of this, MBRs isolated from cancer cells can promote tumorigenic properties in non-transformed cells (Rai et al., 2021). Finally, some midbody proteins, including CIT-K and its partner KIF14, have been linked to brain development and microcephaly (Siskos et al., 2021). Despite the evidence of the involvement of the midbody in these important processes, our understanding of the mechanisms that regulate its formation, functions and inheritance is very limited. Importantly, initial studies have revealed that phosphorylation plays a key role in regulating the activity, function and associations of midbody proteins in both time and space (D'Avino and Capalbo, 2016). For example, the activity and interactions of the centralspindlin complex are regulated by complex changes in its phosphorylation status mediated by CDK1, AURKB, Plk1 and PP1 (Adriaans et al., 2019; Basant et al., 2015; Burkard et al., 2009; Capalbo et al., 2019; Douglas et al., 2010; Mishima et al., 2004; Wolfe et al., 2009). In addition, we have previously described a cross-regulation mechanism between AURKB and CIT-K that controls the assembly of the midbody by temporally regulating the association of CIT-K with both the MKLP1 centralspindlin component and the CPC (McKenzie et al., 2016). Together, these studies highlighted that the assembly and functions of this important organelle are regulated by complex and interconnected phosphorylation events that have yet to be fully understood.

Here, we report a novel regulatory mechanism among mitotic kinases important for the formation and stability of the midbody. We found that CDK1 and AURKB regulate CIT-K distribution and association with its partners by phosphorylating two distinct residues located adjacent to or within the coiled coil domain of CIT-K. Analyses using phospho-specific antibodies and phospho-mimetic and non-phosphorylatable mutants indicated that perturbing these phosphorylation events leads to abnormal midbody formation, accumulation of post-mitotic MBRs, and affects the interaction of CIT-K with its midbody partners. Together, our findings indicate that regulation of CIT-K by CDK1 and AURKB temporally regulates the association of CIT-K with its partners AURKB, centralspindlin and KIF14, in order to finely dictate midbody assembly, stability and inheritance.

## RESULTS

### CIT-K is phosphorylated by CDK1 and AURKB at multiple residues

**Fig. 1.**
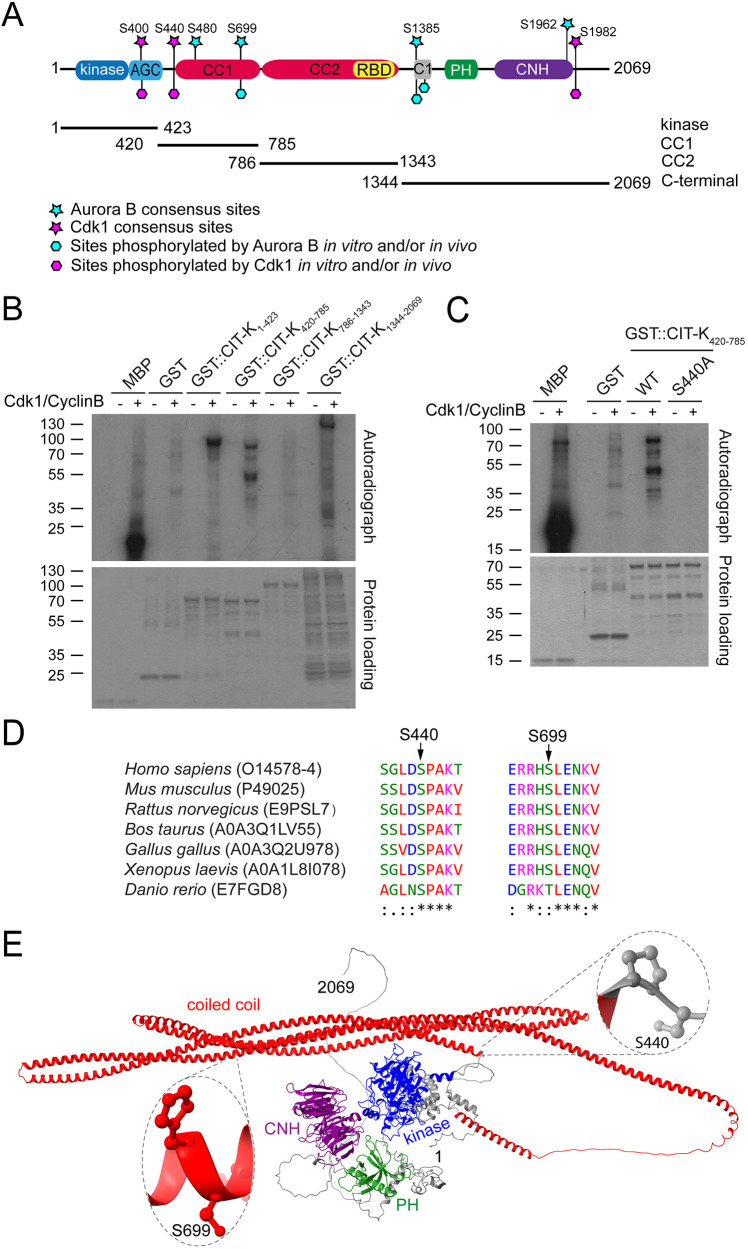
**CDK1 and AURKB phosphorylate distinct CIT-K residues.** (A) Schematic diagram illustrating the protein domains of CIT-K. The positions of the different CIT-K fragments used for the *in vitro* phosphorylation assays are also indicated. CC1 and CC2 indicate the fragments encompassing the first and second coiled coil regions; AGC, AGC-kinase C-terminal domain; RBD, Rho-binding domain; C1, cysteine-rich motif; PH, pleckstrin homology domain; CNH, Citron–Nik1 homology domain. (B) GST-tagged CIT-K fragments, GST alone or the positive control myelin basic protein (MBP) were incubated with (+) or without (−) recombinant CDK1–cyclin B complexes in the presence of [γ-32P] ATP. The reactions were separated by SDS-PAGE, and the gel stained with Coomassie Blue to check the protein loading (shown at the bottom). The gel was then transferred onto a nitrocellulose membrane and exposed to X-ray films. (C) GST-tagged wild-type (WT) CIT-K_420–785_ fragment, its non-phosphorylatable mutant S440A, GST alone or the positive control myelin basic protein (MBP) were incubated with (+) or without (−) recombinant CDK1–cyclin B complexes, in the presence of [γ-32P] ATP. The reactions were separated by SDS-PAGE, and the gel stained with Coomassie Blue to check the protein loading (shown at the bottom). The gel was then transferred onto nitrocellulose membrane and exposed to X-ray films. In B,C, the numbers on the left indicate the sizes in kDa of the molecular mass marker. Images in B,C represent two independent experiments. (D) The amino acid sequences containing the S440 and S699 residues of human CIT-K and its orthologues in other vertebrate species were aligned using the Muscle algorithm (Edgar, 2004). UniProt accession IDs are given in parentheses. Amino acids are colored according to their chemical properties: green, polar; red, non-polar; blue, acidic; magenta, basic. (E) Schematic diagram illustrating the CIT-K structure predicted by AlphaFold 3. The kinase, CC, PH and CNH domains are colored as in A. The positions of S440 and S699 are indicated and magnified.

In our previous proteomics studies of CIT-K protein–protein interaction networks (interactomes) during mitosis (Capalbo et al., 2019; McKenzie et al., 2016), we identified several phosphorylated serine (S) and threonine (T) residues that matched the consensus sequences for CDK1 and AURKB (Fig. 1A). We previously confirmed that S699 and a stretch of two serine and one threonine residues at positions 1385–1387 were phosphorylated by AURKB *in vitro* (McKenzie et al., 2016). We found that the fragments containing potential CDK1 phosphorylation sites, encompassing the kinase domain (CIT-K_1–423_, S400), the first coiled coil region CC1 (CIT-K_420–785_, S440) and the C-terminal region (CIT-K_1344–2060_, S1982), were phosphorylated *in vitro* by CDK1 (Fig. 1B). The CC1 domain has been shown to interact with the kinesins KIF14 and MKLP1 (Bassi et al., 2013; Gruneberg et al., 2006; Watanabe et al., 2013) and we previously reported that phosphorylation of S699 by AURKB regulates CIT-K localization and its association with MKLP1 and the CPC (McKenzie et al., 2016). Therefore, we decided to further investigate the role of S440 phosphorylation in detail, also in relation to AURKB-mediated phosphorylation of S699. First, we confirmed that S440 is a direct CDK1 target residue because a fragment containing a serine to alanine mutation at this residue (S440A) was no longer phosphorylated by CDK1 *in vitro* (Fig. 1C). Both S440 and S699 are conserved in vertebrates (Fig. 1D). We generated a predicted structure of the longest and only CIT-K isoform that contains S699 (UniProt ID O14578-4) using AlphaFold 3 (Abramson et al., 2024). This predicted structure indicated that CIT-K forms a folded protein with the N-terminal kinase domain positioned very close to the C-terminal pleckstrin homology (PH) and Citron–Nik1 homology (CNH) domains (Fig. 1E; Fig. S1). Interestingly, S440 and S699 are exposed and easily accessible, and located in two structurally separated regions (Fig. 1E). In particular, S440 lies in a predicted disordered region just upstream of the CC1 domain, whereas S699 is located within the CC1 domain (Fig. 1A,E).

These results indicate that CIT-K is phosphorylated by CDK1 and AURKB at two distinct and evolutionarily conserved serine residues located adjacent or within the first coiled coil domain, which mediates the interaction of CIT-K with KIF14 and MKLP1 (Bassi et al., 2013; Gruneberg et al., 2006; Watanabe et al., 2013).

### S440 and S699 phosphorylations display distinct temporal profiles

**Fig. 2.**
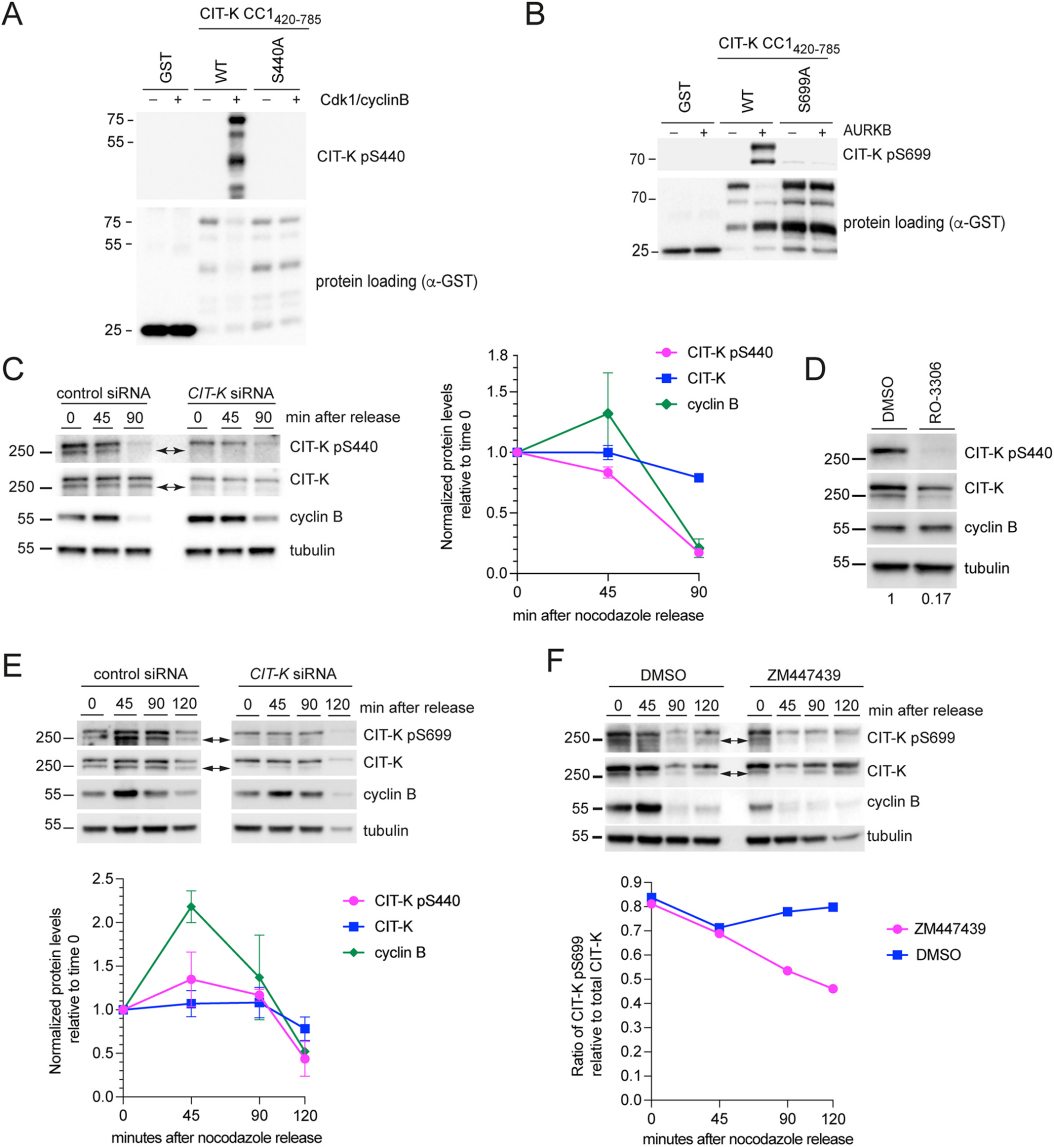
**Temporal profiles of S440 and S699 phosphorylation.** (A) GST-tagged WT CIT-K_420–785_ fragment, its non-phosphorylatable mutant S440A and GST alone were incubated with (+) or without (−) recombinant CDK1/cyclin B. The reactions were then separated by SDS-PAGE and analyzed by western blotting to detect phosphorylated S440 (pS440) and GST. Images represent three independent experiments. (B) GST-tagged WT CIT-K_420–785_ fragment, its non-phosphorylatable mutant S699A and GST alone were incubated with (+) or without (−) recombinant Aurora B (AURKB). The reactions were then separated by SDS PAGE and analyzed by western blotting to detect phosphorylated S699 (pS699) and GST. Images represent three independent experiments. (C) HeLa Kyoto cells stably expressing GFP-tagged CIT-K were treated with siRNAs directed against either a random sequence (control) or the 3′ UTR of *CIT-K*. During RNAi incubation, cells were synchronized by thymidine/nocodazole block and then collected at the indicated time points after nocodazole release. Proteins were extracted and analyzed by western blotting to detect CIT-K, CIT-K pS440, cyclin B and tubulin as the loading control. The arrows indicate endogenous untagged CIT-K. The graph on the right shows the quantification of protein levels, normalized to tubulin and relative to the 0 min time point, from at least two different western blots. Bars indicate mean±s.e.m. (D) HeLa Kyoto cells stably expressing GFP-tagged CIT-K were synchronized by thymidine/nocodazole block, released for 45 min and incubated for an additional 30 min with either the CDK1 inhibitor RO-3306 or its solvent DMSO as control. Proteins were extracted and analyzed by western blotting to detect CIT-K, CIT-K pS440, cyclin B and tubulin as the loading control. The numbers at the bottom indicated the quantification of the normalized levels of CIT-K pS440 relative to those of CIT-K. Images represent two independent experiments. (E) HeLa Kyoto cells stably expressing GFP-tagged CIT-K were treated with siRNAs directed against either a random sequence (control) or the 3′ UTR of *CIT-K*. During RNAi incubation, cells were synchronized by thymidine/nocodazole block and then collected at the indicated time points after nocodazole release. Proteins were extracted and analyzed by western blotting to detect CIT-K, CIT-K pS699, cyclin B and tubulin as the loading control. The arrows indicate endogenous untagged CIT-K. The graph below shows the quantification of protein levels, normalized to tubulin and relative to the 0 min time point, from at least two different western blots. Bars indicate mean±s.e.m. (F) HeLa Kyoto cells stably expressing GFP-tagged CIT-K were synchronized by thymidine/nocodazole block, released and incubated with either the AURKB inhibitor ZM447439 or its solvent DMSO. Cells were collected at the indicated time points and proteins were extracted and analyzed by western blotting to detect CIT-K, CIT-K pS699, cyclin B and tubulin as the loading control. The arrows indicate endogenous untagged CIT-K. The graph below shows the quantification of protein levels, normalized to tubulin and relative to the 0 min time point, of the ratio of CIT-K pS699 versus endogenous CIT-K. The numbers on the left of blots indicate the sizes in kDa of the molecular mass marker. This experiment was undertaken once.

**Fig. 3.**
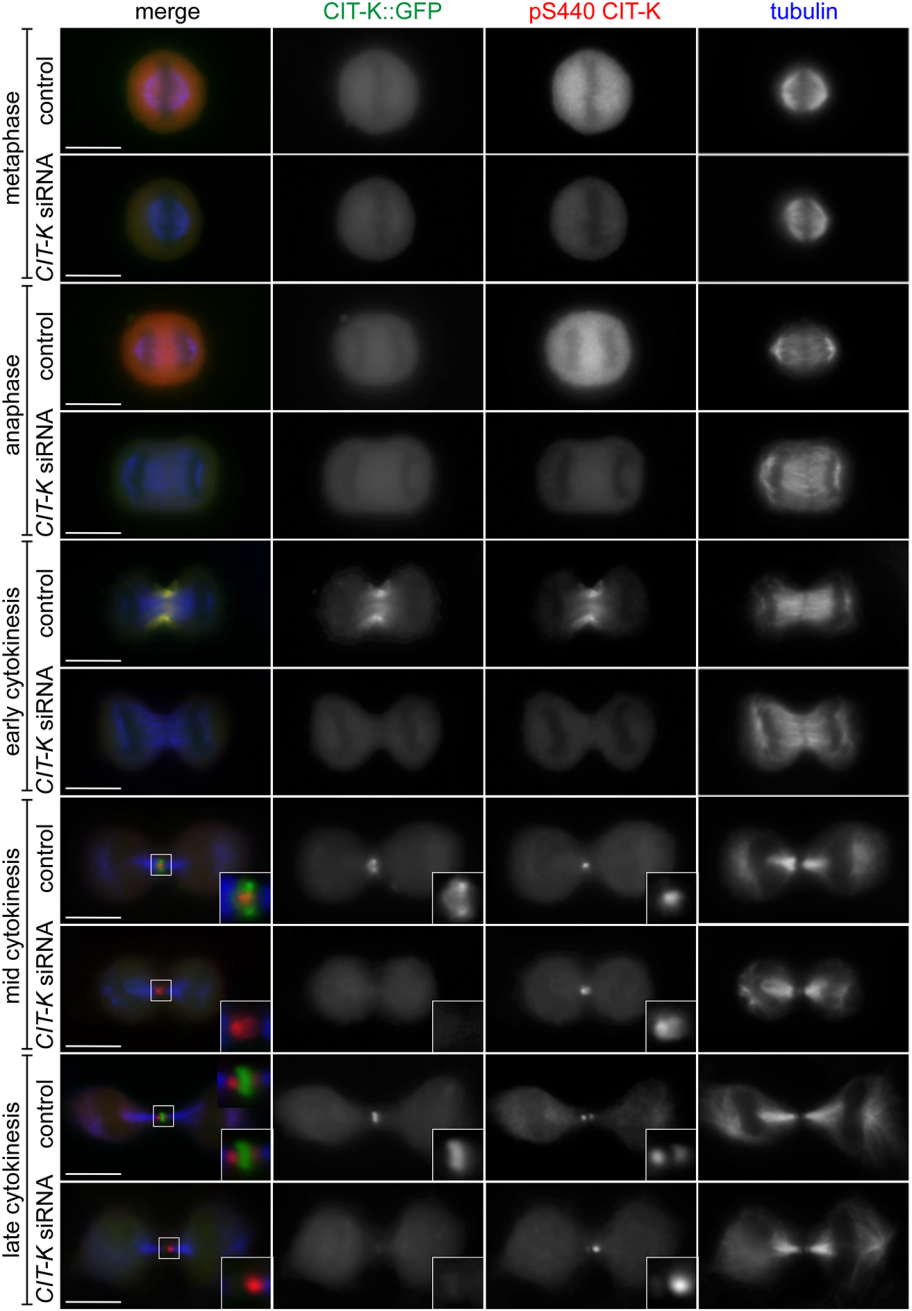
**Localization of CIT-K phosphorylated at S440 during cell division.** HeLa Kyoto cells stably expressing GFP-tagged CIT-K were treated with siRNAs directed against either a random sequence (control) or the coding region of *CIT-K* and, after 48 h, were fixed and stained to detect CIT-K::GFP (green), CIT-K pS440 (red) and tubulin (blue). The shape and thickness of microtubule bundles at the intercellular bridge were used as criteria to stage telophase cells. Insets show a 3× magnification of the midbody. Images represent three independent experiments. Scale bars: 10 µm.

To analyze the temporal and spatial profiles of S440 and S699 phosphorylation, we generated antibodies directed against peptides phosphorylated at either S440 (pS440) or S699 (pS699). These antibodies specifically recognized the CIT-K_420–785_ fragment phosphorylated *in vitro* by CDK1 and AURKB, and not fragments in which the S440 and S669 residues were mutated to alanine (Fig. 2A,B). We then analyzed the phosphorylation profiles of pS440 and pS699 by western blotting analyses using extracts from cells synchronized at different mitotic stages through thymidine/nocodazole block and release. To assess the specificity of the two phospho-antibodies, we used a HeLa cell line expressing a *CIT-K::GFP* transgene (Capalbo et al., 2019; McKenzie and D'Avino, 2016) and extracted proteins from cells depleted of endogenous CIT-K by using an siRNA directed against the 3′ untranslated region (UTR) of CIT-K, which is not present in the *CIT-K::GFP* transgene. Western blot analysis showed that both phospho-antibodies recognized two bands in control cells and that the faster migrating band, corresponding to endogenous CIT-K, was absent in extracts from cells treated with the 3′ UTR *CIT-K* siRNA (Fig. 2C,E). These results indicated that the antibodies specifically recognize CIT-K phosphorylated at S440 and S699. We found that the CIT-K pS440 signal decreased 90 min after exit from mitosis when compared to the total CIT-K signal, parallel to cyclin B degradation (Fig. 2C). In addition, the CIT-K pS440 signal was markedly reduced after treatment with the CDK1 inhibitor RO-3306 (Fig. 2D), confirming that pS440 is phosphorylated by CDK1 in cells. By contrast, the pS699 signal showed a less marked decline compared to the total CIT-K signal, continuing to be present 120 min after release from nocodazole (Fig. 2E). CIT-K pS699 levels reduced after treatment with the AURKB inhibitor ZM447439 (Fig. 2F), further confirming that S699 is phosphorylated by AURKB in cells. Immunofluorescence analysis indicated that CIT-K pS440 was found diffuse in the cytoplasm in metaphase and anaphase, accumulated at the cleavage furrow in early telophase, and then, after completion of furrow ingression, it localized to the midbody core and arms instead of the normal localization of CIT-K to midbody ring (Fig. 3) (Bassi et al., 2013; Capalbo et al., 2019; D'Avino, 2017). The pS440 signals in metaphase, anaphase and early cytokinesis colocalize with those of GFP::CIT-K and are reduced or absent after *CIT-K* RNAi, indicating that they specifically detect pS440 CIT-K (Fig. 3). By contrast, the pS440 signals to the midbody core and arms in mid and late cytokinesis cells did not colocalize with CIT-K::GFP and did not always disappear after *CIT-K* RNAi (Fig. 3), indicating that they might not be specific for CIT-K. However, considering that antibody staining is more sensitive than GFP fluorescence, we cannot exclude that this signal might represent a relatively small but particularly stable population of CIT-K pS440. Unfortunately, the CIT-K pS669 antibody failed to detect any specific signal that was reduced or eliminated after *CIT-K* RNAi, indicating that this phospho-antibody is not suitable for immunofluorescence experiments (Fig. S2).

Our findings indicate that CIT-K is phosphorylated at S440 and S699 in early mitotic stages, but whereas S440 is de-phosphorylated after anaphase onset, S699 remains phosphorylated in anaphase and telophase. These results are consistent with the evidence that CDK1 activity declines after cyclin B degradation in anaphase, whereas AURKB remains active in cytokinesis (D'Avino and Capalbo, 2015, 2016; Lindon et al., 2015; Wieser and Pines, 2015).

### Phosphorylation at S440 regulates midbody formation and stability

**Fig. 4.**
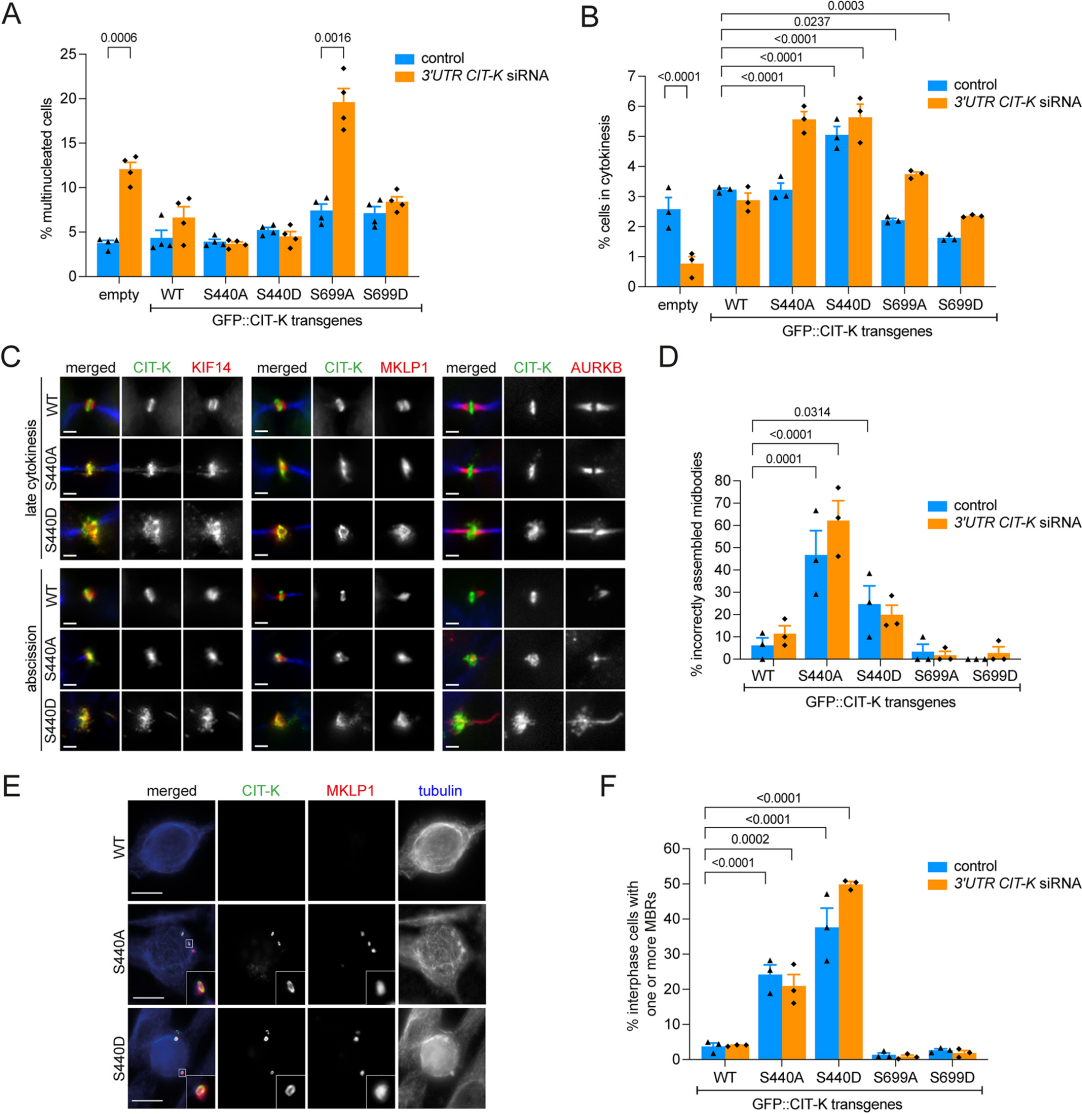
**CIT-K phosphorylation at S440 and S699 regulates midbody structure and stability.** (A,B) HeLa cells expressing the indicated GFP::CIT-K constructs or no transgene (empty) were transfected with either an siRNA directed against either a random sequence (control) or the 3′ UTR of *CIT-K* and, after 48 h, were fixed and stained to detect tubulin and DNA. The samples were then analyzed to quantify the percentage of multinucleated cells (A) and daughter cells linked by an intercellular bridge (cells in cytokinesis) (B). More than 1000 cells were counted in *n*≥3 independent experiments. Bars indicate mean±standard errors. Significant *P*-values are indicated at the top of the graph (unpaired one-tailed *t*-test in A and two-way ANOVA in B). (C) HeLa cells expressing the indicated GFP::CIT-K constructs were treated with siRNA directed against the 3′ UTR of *CIT-K* and, after 48 h, were fixed and stained to detect CIT-K (green), tubulin (blue) and KIF14, MKLP1 or AURKB (red). The shape and thickness of microtubule bundles at the intercellular bridge were used as criteria to stage cells. Scale bars: 2 µm. (D) Quantification of cells in late cytokinesis showing disorganized and/or fragmented midbodies from the experiments shown in A–C. More than 500 cells were counted in *n*≥3 independent experiments. Bars indicate mean±standard errors. Significant *P*-values are indicated at the top of the graph (two-way ANOVA). (E) HeLa cells expressing the indicated GFP::CIT-K constructs were treated with siRNA directed against the 3′ UTR of *CIT-K* and, after 48 h, were fixed and stained to detect CIT-K (green), tubulin (blue) and MKLP1 (red), and then analyzed to quantify the number of midbody remnants (MBRs). Scale bars: 10 µm. (F) Quantification of interphase cells from the experiments shown in E containing one or more MBRs. More than 500 cells were counted in *n*≥3 independent experiments. Bars indicate mean±standard errors. Significant *P*-values are indicated at the top of the graph (two-way ANOVA).

To investigate the role of S440 and S699 phosphorylation during cell division, we generated HeLa cell lines stably expressing doxycycline-inducible transgenes expressing GFP-tagged CIT-K wild-type (WT) or with phospho-mimetic (S to D) or phospho-dead (S to A) mutations at these residues. We selected individual clones that expressed the transgenes at levels that were most similar to those of endogenous CIT-K after testing different concentrations of doxycycline (Fig. S3). We analyzed the distribution of these mutant transgenes in both control cells and cells depleted of the endogenous CIT-K using an siRNA targeting the 3′ UTR region of the gene, which is not present in the transgenes. Both mutant proteins localized to the midbody in late cytokinesis (Fig. S4). However, S440 mutant CIT-K proteins often failed to assemble in the ring-like structure typical of WT CIT-K and showed excessive accumulation to the midbody in very late cytokinesis, just before abscission, a phenotype that was more penetrant for the S440D mutant (Fig. S4B,C). We then tested the ability of these mutants to rescue the multinucleation (a readout of cytokinesis failure) caused by RNAi depletion of endogenous CIT-K and found that only the S699A mutant failed to rescue multinucleation (Fig. 4A). We also determined the frequency of daughter cells linked by an intercellular bridge (cells in cytokinesis) as a readout for possible abscission delays. Depletion of CIT-K in the parental cells led to a decrease of cells in cytokinesis (Fig. 4B), which is consistent with the established role of CIT-K in midbody formation (Bassi et al., 2013; McKenzie et al., 2016). Both S440A and S440D mutants showed a significant increase of cells in cytokinesis, which, in the case of S440D, also occurred in the presence of endogenous CIT-K, indicating a dominant effect (Fig. 4B). More modest increases in cells in cytokinesis were found with S669A and S669D mutants after 3′ UTR *CIT-K* siRNA depletion, but they were not significant when compared to those seen for the WT transgene (Fig. 4B). We also found that the distribution of S440A and S440D mutant proteins to the midbody appeared disorganized and fragmented in late cytokinesis and post abscission (Fig. 4C,D). Co-staining with the midbody components and CIT-K interactors AURKB, KIF14 and MKLP1 confirmed that these represented entire midbodies and not just aggregates of the GFP::CIT-K mutant proteins. Notably, the midbody distribution of these three CIT-K interactors was altered in cells expressing S440A and S440D mutants (Fig. 4C). In particular, AURKB appeared to unusually persist at the midbody after abscission in both cell lines and its signal was also abnormally observed at the midbody core, where it partially colocalized with CIT-K mutant proteins (Fig. 4C). These incorrectly assembled midbodies were more frequent in cells expressing the S440A mutant, but more severe in cells expressing the S440D mutant (Fig. 4C,D). Finally, we found that interphase cells expressing the S440A and S440D CIT-K mutant proteins contained more MBRs (Fig. 4E,F). Midbody fragmentation and increased MBRs were not observed in cells expressing S699A or S699D mutants (Fig. 4D,F).

Taken together, these results indicate that S440 phosphorylation has a more relevant role on midbody formation and stability, and in turn on abscission, than phosphorylation at S699, which instead seems to be more important for CIT-K localization and successful cytokinesis.

### Phosphorylation at S440 and S699 regulates CIT-K association with its midbody partners

**Fig. 5.**
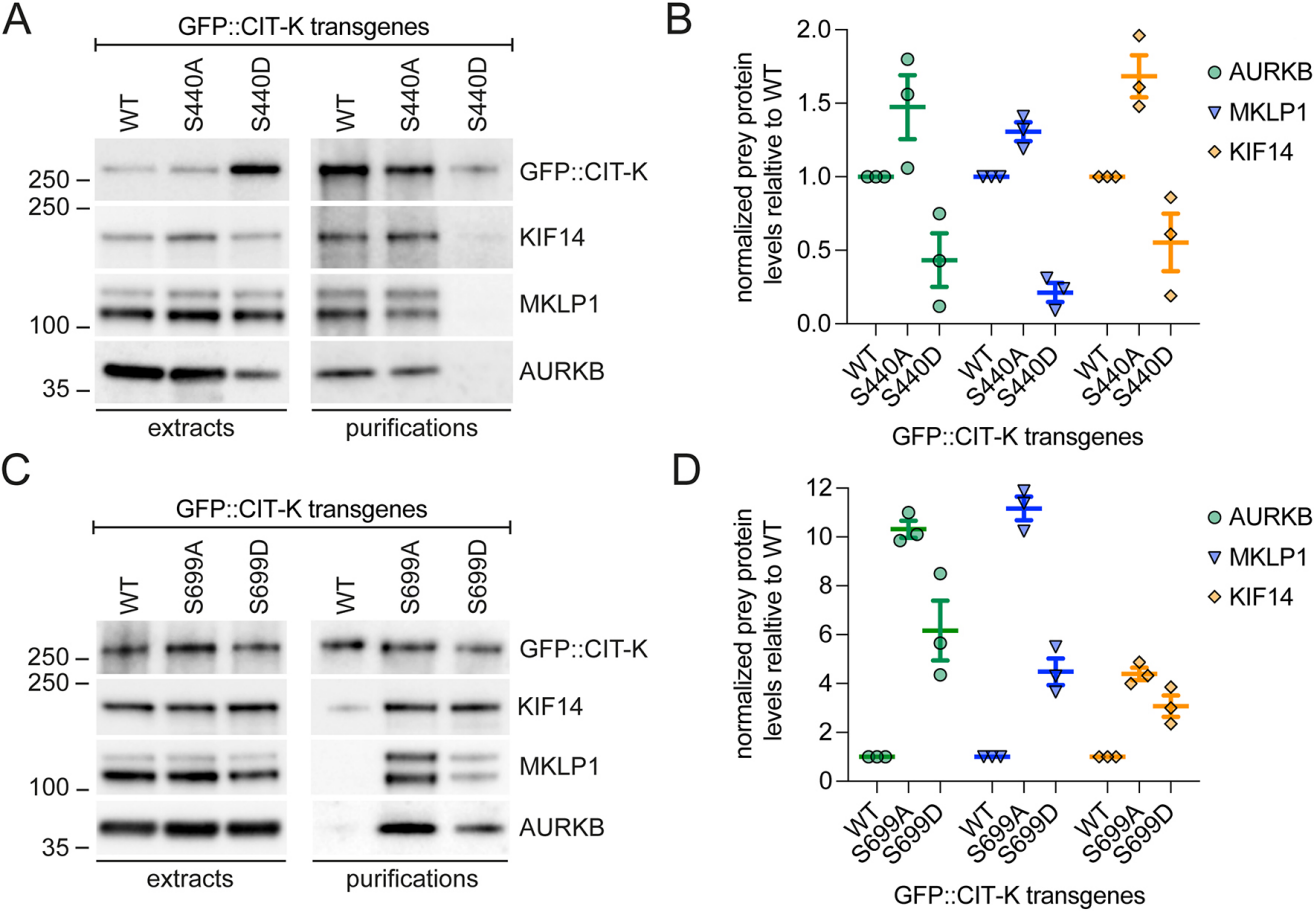
**Phosphorylation at S440 and S699 affects CIT-K interaction with its midbody partners.** (A) HeLa cells stably expressing the indicated GFP-tagged CIT-K transgenes were treated with an siRNA directed against the 3′UTR of *CIT-K*. During RNAi incubation, cells were synchronized by thymidine/nocodazole block and then collected 90 min after nocodazole release. Protein extracts were used in a GFP pull-down assay and then analyzed by western blotting to detect the proteins indicated at the right. The numbers on the left indicate the sizes in kDa of the molecular mass marker. (B) Quantification and comparison of the levels of purified prey proteins from experiments as shown in A. The levels of captured prey proteins were first normalized to the intensity levels of their relative GFP::CIT-K baits, and then compared to the normalized levels of prey proteins captured by the GFP::CIT-K wild-type (WT) bait. Data are from three independent experimental replicates. Bars indicate mean±s.e.m. (C) HeLa cells stably expressing the indicated GFP-tagged CIT-K transgenes were treated with an siRNA directed against the 3′UTR of *CIT-K*. During RNAi incubation, cells were synchronized by thymidine/nocodazole block and then collected 90 min after nocodazole release. Protein extracts were used in a GFP pull-down assay and then analyzed by western blotting to detect the proteins indicated at the right. The numbers on the left indicate the sizes in kDa of the molecular mass marker. (D) Quantification and comparison of the levels of purified prey proteins from experiments as shown in C. The levels of captured prey proteins were first normalized to the intensity levels of their relative GFP::CIT-K baits, and then compared to the normalized levels of prey proteins captured by the GFP::CIT-K wild-type (WT) bait. Data are from three independent experimental replicates. Bars indicate mean±s.e.m.

To gain insights into the possible underlying molecular mechanisms of CIT-K regulation via S440 and S699 phosphorylation, we investigated the ability of the S440 and S699 phospho-dead and phospho-mimetic mutants to interact with known CIT-K midbody partners, namely, AURKB, KIF14 and MKLP1, by immunoprecipitation using proteins extracted from cells synchronized in telophase and depleted of endogenous CIT-K. The S440A phospho-dead mutation slightly increased the association of CIT-K::GFP with its midbody partners (Fig. 5A,B). By contrast, the S440D mutation considerably reduced the ability of CIT-K to pull down any of its partners (Fig. 5A,B). In agreement with our previous findings (McKenzie et al., 2016), the S699A mutation enhanced the association of CIT-K with all three midbody partners, although much more strongly with AURKB and MKLP1 (Fig. 5C,D). The S699D mutant associated with the three partners less efficiently than S699A, but more strongly than the WT protein (Fig. 5C,D). Please note that the signals of AURKB and MKLP1 in the WT GFP::CIT-K purifications are less intense than in the experiment in Fig. 5A because of shorter exposure times. Longer exposures confirmed the presence of MKLP1 and AURKB in these purifications (Fig. S5). This is consistent with our previously published experiment using a different cell line expressing the S699A mutant (McKenzie et al., 2016).

**Fig. 6.**
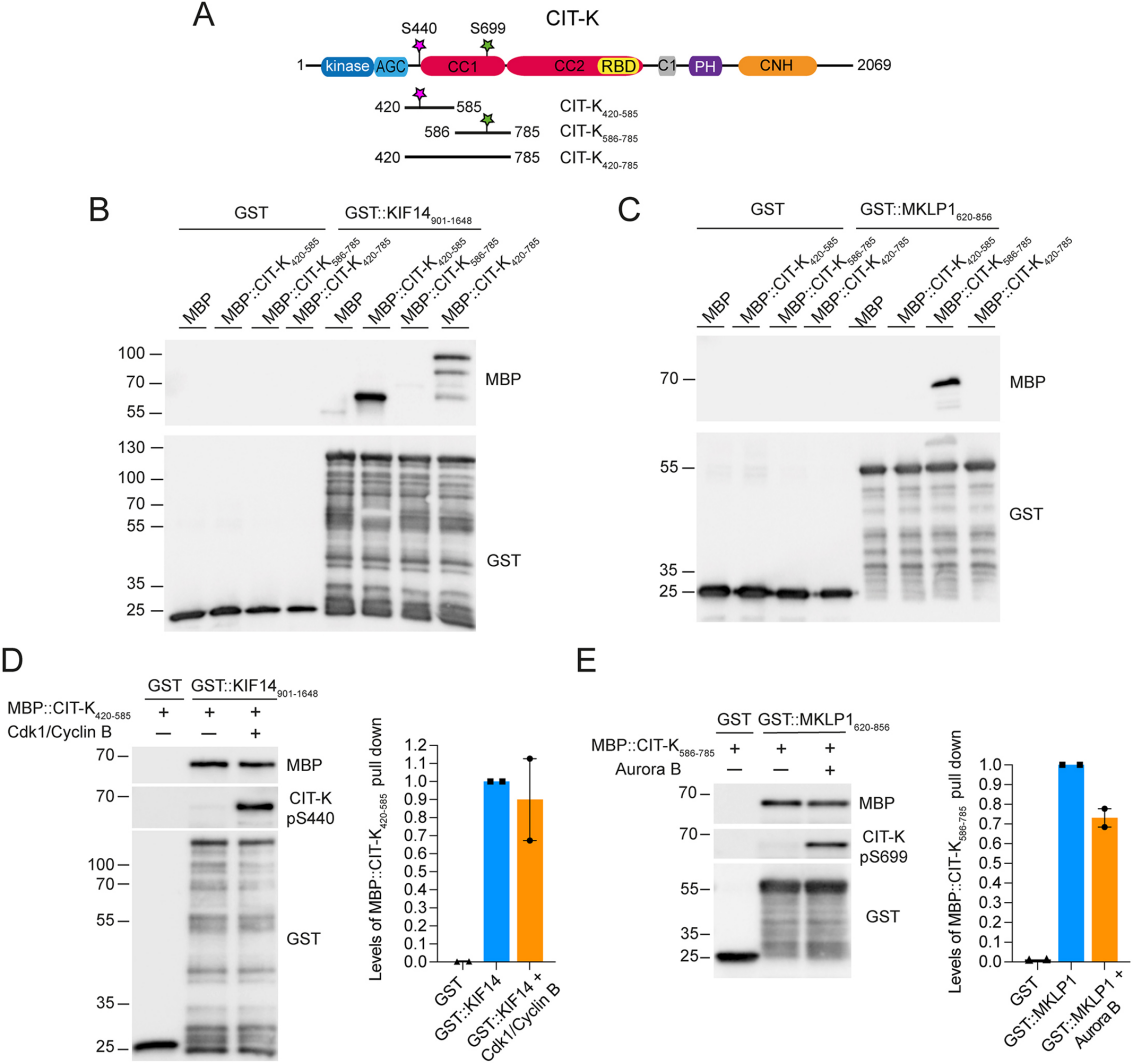
**KIF14 and MKLP1 bind to distinct regions within the first coiled coil domain of CIT-K.** (A) Schematic diagram illustrating the protein domains of CIT-K. The S440 and S699 residues and the different CIT-K fragments used for the *in vitro* pull down assays are indicated. CC1 and CC2 indicate the fragments encompassing the first and second coiled coil regions; AGC, AGC-kinase C-terminal domain; RBD, Rho-binding domain; C1, cysteine-rich motif; PH, pleckstrin homology domain; CNH, Citron–Nik1 homology domain. (B) The GST::KIF14_901–1648_ fragment and GST alone were incubated with the indicated maltose binding protein (MBP)-tagged CIT-K fragments, and then pulled down using glutathione beads. The pull downs were then analyzed by western blotting. (C) The GST::MKLP1_620–856_ fragment and GST alone were incubated with the indicated MBP-tagged CIT-K fragments, and then pulled down using glutathione beads. The pull downs were then analyzed by western blotting. (D) The MBP::CIT-K_420–585_ fragment was incubated with (+) or without (−) recombinant CDK1/cyclin B and then used in pull down assays with the GST::KIF14_901–1648_ fragment and GST alone. The pull downs were analyzed by western blotting using antibodies against MBP, CIT-K pS440 and GST. The graph on the right shows the quantification of the protein levels of MBP::CIT-K_420–585_, normalized to GST::KIF14_901–1648_ or GST alone, and relative to unphosphorylated (−) MBP::CIT-K_420–585_. Bars indicate mean±standard errors. (D) The MBP::CIT-K_586–785_ fragment was incubated with (+) or without (−) recombinant Aurora B (AURKB) and then used in pull down assays with the GST::MKLP1_620–856_ fragment and GST alone. The pull downs were then analyzed by western blotting using antibodies against MBP, CIT-K pS699 and GST. The graph on the right shows the quantification of the protein levels of MBP::CIT-K_586–785_, normalized to GST::MKLP1_620–856_ or GST alone, and relative to unphosphorylated (−) MBP::CIT-K_586–785._ Bars indicate mean±standard errors. The numbers on the left of western blots in B–E indicate the sizes in kDa of the molecular mass markers.

CIT-K directly binds to KIF14 and MKLP1 through its CC1 domain (Bassi et al., 2013; McKenzie et al., 2016; Watanabe et al., 2013), whereas AURKB and other CPC components bind to the CNH domain (McKenzie et al., 2016). As both S440 and S699 are located near or within the CC1 region, we investigated whether the ability of CIT-K to bind to KIF14 and/or MKLP1 *in vitro* was affected by phosphorylation at these two residues. We divided the fragment containing the CC1 domain, CIT-K_420–785_, into two smaller peptides, CIT-K_420–585_ and CIT-K_586–785_, the first containing the S440 residue and the other containing S699 (Fig. 6A). These peptides were tagged with maltose-binding protein (MBP), expressed and purified from bacteria, and then tested in an *in vitro* pull down assay for their ability to bind to bacterially purified GST-tagged KIF14_901–1648_ and MKLP1_620–856_ peptides, known to directly interact with CIT-K (Bassi et al., 2013; Watanabe et al., 2013). GST::KIF14_901–1648_ specifically pulled down the CIT-K_420–585_ fragment containing S440 (Fig. 6B), whereas GST::MKLP1_620–856_ specifically pulled down the other peptide, CIT-K_586–785,_ containing S699 (Fig. 6C). We then tested whether phosphorylation at S440 or S699 affected the binding of these CIT-K fragments to KIF14 or MKLP1. S440 phosphorylation did not appear to affect the binding of CIT-K_420–585_ to KIF14 (Fig. 6D), whereas S699 phosphorylation consistently reduced the association of CIT-K_586–785_ to MKLP1 *in vitro* (Fig. 6E).

Taken together, our findings indicate that the phosphorylation at S440 and S699 reduces the ability of CIT-K to interact with its midbody partners AURKB, KIF14 and MKLP1 in cells. *In vitro* binding assays indicate that S440 phosphorylation does not affect the binding of CIT-K to KIF14, while whereas phosphorylation causes a reduction in the binding of CIT-K to MKLP1.

## DISCUSSION

CIT-K is one of the key factors that control midbody assembly and architecture in late cytokinesis (D'Avino, 2017). We previously showed that CIT-K is necessary to maintain the orderly arrangement of midbody proteins and that cross-regulation between CIT-K and AURKB regulates midbody formation (Bassi et al., 2013; McKenzie et al., 2016). More specifically, AURKB phosphorylation of CIT-K at S699 prevents its accumulation at the spindle midzone in early cytokinesis by inhibiting the association of CIT-K with MKLP1 and the CPC (McKenzie et al., 2016). Here, we expand these previous findings by showing that CIT-K is also phosphorylated by CDK1 at S440 and that phosphorylation by CDK1 and AURKB regulates CIT-K distribution and its association with midbody partners (Figs 4 and 5) (McKenzie et al., 2016). The temporal profiles of S440 and S699 phosphorylation reflect the activity of the kinases responsible for their phosphorylation. Both residues are phosphorylated in early mitosis, but S440 is dephosphorylated earlier, at anaphase onset, in parallel with CDK1 inactivation (Figs 2 and 3). Our functional analyses using phospho-mimetic and non-phosphorylatable mutants indicate that these phosphorylation events have both convergent and distinct effects. They confirm and expand our previous findings that S699 phosphorylation by AURKB inhibits the association of CIT-K with some of its midbody partners (Fig. 5) (McKenzie et al., 2016). This mechanism appears necessary for regulating CIT-K function and cytokinesis progression because the S699A mutant failed to rescue cytokinesis failure caused by CIT-K depletion (Fig. 4A). Results from the experiments with S440 phospho-mutants indicate that CDK1 phosphorylation also inhibits the interaction of CIT-K with its midbody partners, even more efficiently than AURKB phosphorylation (Fig. 5). Taken together, these findings suggest that CDK1 and AURKB phosphorylations regulate CIT-K interactions to accurately set the timing of midbody formation. They might also prevent premature binding of CIT-K to MKLP1 in early cytokinesis, which might interfere with centralspindlin clustering and central spindle assembly (Douglas et al., 2010; Hutterer et al., 2009). However, S440 mutants rescued CIT-K depletion (Fig. 4A) and did not cause incorrect localization of CIT-K to the central spindle in early cytokinesis like the S669A mutant (McKenzie et al., 2016). One possible explanation for this discrepancy is that in early mitosis, AURKB phosphorylation is sufficient for regulating CIT-K in the absence of S440 phosphorylation. To test this hypothesis, we considered the possibility of studying the effects of S440 and S699 mutant combinations, but unfortunately our attempts to generate cell lines expressing any of the possible double-mutant combinations were unsuccessful. In addition, results from these experiments might be difficult to interpret.

The S440 and S699 residues are located in two distinct structural regions that specifically bind to KIF14 or MKLP1 *in vitro* (Figs 1 and 6). The results of these *in vitro* binding assays are only partially consistent with the outcome of the immunoprecipitation assays in cells expressing the S440 and S699 phospho-mutants (Fig. 5). For example, the S440 phospho-mimetic mutant drastically reduced the association of CIT-K with KIF14, AURKB and MKLP1 in cells (Fig. 5), but the peptide containing this residue bound only to KIF14 *in vitro*, and S440 phosphorylation does not appear to affect this binding (Fig. 6). Similarly, the S699 phospho-dead mutant interacted much more efficiently with AURKB, KIF14 and MKLP1 (Fig. 5), but the fragment harboring this serine bound specifically to MKLP1 *in vitro*, and S699 phosphorylation only slightly reduced this interaction (Fig. 6). Taken together, these findings indicate that phosphorylation at these two residues in cells must have a more widespread effect on the ability of CIT-K to interact with its partners than just local inhibition. For example, these phosphorylations could have knock-on effects on sequential binding events and/or on overall CIT-K structure.

Our findings indicate that perturbing S440 phosphorylation has an impact on midbody formation and stability (Fig. 4). Cells expressing phospho-dead and phospho-mimetic S440 CIT-K mutants presented disorganized and fragmented midbodies, characterized by incorrect distribution of CIT-K and its partners, AURKB, KIF14 and MKLP1 (Fig. 4C,D). These defects in midbody formation were accompanied by an increase in the frequency of cells in late cytokinesis (Fig. 4B), which most likely reflects a delay in abscission as a consequence of incorrectly assembled midbodies. Finally, the increase of MBRs in interphase cells expressing S440 phospho-mutant proteins (Fig. 4E,F) indicates that MBRs are more stable and persist for longer. Notably, all these phenotypes were not observed in cells expressing S699 CIT-K mutant proteins. Taken together, these results indicate that S440 phosphorylation has a role in regulating CIT-K functions important for midbody assembly and MBR stability. These observations are unexpected because CDK1 activity and S440 phosphorylation decline in anaphase (Fig. 2C). It is possible that the phospho-mimetic S440D mutation has a dominant-negative effect, because it would keep CIT-K in a pre-anaphase state, thereby sustaining inhibition of CIT-K binding to its partners and affecting proper midbody formation. Expression of the phospho-dead S440A mutant also affected midbody formation, although with less severe phenotypes (Fig. 4C). Possible explanations are that the S440A mutation does not act as expected or that inhibiting S440 phosphorylation before anaphase affects CIT-K function in cytokinesis. In addition, we cannot exclude the possibility that S440 is phosphorylated in late cytokinesis by either CDK1 or another kinase. This would be consistent with the evidence that cyclin B2 has been shown to accumulate at the intercellular bridge in late cytokinesis (Mathieu et al., 2013), CDK1 was identified in two independent proteomic analyses of the midbody (Capalbo et al., 2019; Skop et al., 2004), and CDK1 inhibitors delay abscission (Mathieu et al., 2013). Furthermore, it might explain the detection of CIT-K pS440 persistent signals at the midbody core and arms in cells in late cytokinesis (Fig. 3). However, our western blot analyses indicate that this pool of phosphorylated S440 CIT-K at the midbody would not be very abundant (Fig. 2).

In conclusion, here, we present evidence that phosphorylation of CIT-K by CDK1 and AURKB regulates midbody formation and MBR stability by dictating the timing and strength of CIT-K interaction with its partners. These findings not only expand and refine our understanding of the molecular mechanisms that regulate abscission, but can help to unravel how MBR stability is regulated, which, in turn, might lead to further insights into the role of MBRs in post-mitotic events and carcinogenesis.

## MATERIALS AND METHODS

### Molecular biology

The clones containing different CIT-K fragments were generated as previously described (McKenzie et al., 2016). The QuikChange Lightning Site-Directed Mutagenesis Kit (Agilent) was used to generate the S440 and S699 phospho-dead (S to A) and phospho-mimetic (S to E) CIT-K mutants. For inducible expression of GFP-tagged CIT-K constructs in HeLa T-REx cells (Thermo Fisher Scientific), DNA fragments were cloned into the pcDNA FRT⁄TO vector (Thermo Fisher Scientific). Sequences of all DNA constructs were verified by sequencing (Source BioScience).

### *In vitro* phosphorylation assay

DNA fragments coding for CIT-K fragments were generated by PCR and cloned into pDEST15 (Thermo Fisher Scientific) to express N-terminal GST-tagged polypeptides in *Escherichia coli*. Purified GST-tagged CIT-K fragments and myelin basic protein (MBP, Sigma-Aldrich) were incubated with 150 ng of human recombinant CDK1/cyclin B (Thermo Fisher Scientific), 0.1 mM ATP (Sigma-Aldrich), 5 μCi of [γ-^32^P] ATP (6000 Ci/mmol, 10 mCi/ml) (PerkinElmer) and kinase buffer (20 mM HEPES pH 7.5, 2 mM MgCl_2_, 1 mM DTT) in a final reaction volume of 15 μl. After 30 min incubation at 30°C with constant agitation, 15 μl of 2× Laemmli sample buffer (Sigma-Aldrich) was added to stop the reaction. Samples were boiled for 10 min and loaded on a 4–20% Tris-Glycine precast gel (Thermo Fisher Scientific). Gels were stained with Quick Coomassie Stain (Generon) to check the protein loading and then proteins were transferred onto a nitrocellulose membrane using the iBlot Dry Blotting System (Invitrogen). Membranes were exposed to Kodak BioMax XAR Films (Sigma-Aldrich) at −80°C. Uncropped images of the autoradiographs are shown in Fig. S5.

Non-radioactive *in vitro* kinase assays were performed as above, but without [γ-^32^P] ATP and using 150 ng of human recombinant CDK1/cyclin B or 190 ng of human recombinant AURKB (Thermo Fisher Scientific) and ATP at a final concentration of 0.5 mM.

### Cell culture, siRNA transfection, drug treatments and generation of stable cell lines

HeLa Kyoto cells were maintained in Dulbecco's modified Eagle medium (DMEM, Sigma-Aldrich) containing 10% (v/v) fetal bovine serum (Sigma-Aldrich) and 1% (v/v) penicillin/streptomycin (Invitrogen) at 37°C and 5% CO_2_. HeLa T-REx cells (Thermo Fisher Scientific) were cultured in DMEM containing 10% (v/v) fetal bovine serum without tetracycline (FBS-TET, Sigma-Aldrich) and 1% (v/v) penicillin/streptomycin at 37°C and 5% CO_2_. Transfected HeLa T-REx cell lines were maintained in the same medium containing 5 μg/ml blasticidin S and 250 μg/ml hygromycin B (Thermo Fisher Scientific).

For RNAi, the following siRNAs were used: scrambled sequence control, 5′-AACGTACGCGGAATACTTCGA-3′, and 3′ UTR *CIT-K*, 5′-CACACUAUGGAACUCUGCU-3. These were transfected using Lipofectamine RNAiMAX (Thermo Fisher Scientific) following the manufacturer's instructions. These siRNAs have been previously validated for specificity and efficacy (McKenzie et al., 2016).

To generate HeLa T-REx inducible cell lines expressing WT or S440 and S699 mutant CIT-K constructs, 2×10^6^ cells were transfected with 10 μg of the relevant plasmid and the pOG444 plasmid (Thermo Fisher Scientific) in a 7:3 ratio using the Neon Transfection System (Thermo Fisher Scientific) and the following settings: 1005 V, 35 ms, two pulses. After 48 h, cells in 100 mm culture dishes were selected in complete medium containing 5 μg/ml blasticidin S and 250 μg/ml hygromycin B for approximately 2 weeks until colonies became visible. Individual colonies were picked, cultured under resistance and tested for expression of the construct by western blotting and immunofluorescence.

HeLa cells were synchronized in metaphase and telophase by using the thymidine/nocodazole block and release procedure previously described (Capalbo et al., 2019; McKenzie et al., 2016). Cells were first arrested in S phase by the addition of 2 mM thymidine (Sigma-Aldrich) for 19 h, washed twice with phosphate-buffered saline (PBS) and released for 5 h in fresh complete medium. Cells were then cultured for additional 13 h in fresh complete medium containing 50 ng ml^−1^ nocodazole (Sigma-Aldrich) and then harvested by mitotic shake-off. Mitotic cells were washed five times with PBS, released for 90 min in fresh medium and then harvested by centrifugation and frozen in dry ice.

Cells were incubated with 10 µM RO-3306 (Calbiochem) to inhibit CDK1 or with 5 µM ZM447439 (Tocris Biosciences) to inhibit AURKB.

### Fluorescence microscopy

HeLa cells were grown on microscope glass coverslips (Menzel-Gläser) and fixed in either PHEM buffer [60 mM PIPES, 25 mM HEPES pH 7, 10 mM EGTA, 4 mM MgCl_2_, 3.7% (v/v) formaldehyde] for 12 min at room temperature or in ice-cold methanol for 10 min at −20°C. They were then washed three times for 5 min with PBS and incubated in blocking buffer [PBS, 0.5% (v/v) Triton X-100 and 5% (w/v) BSA (Sigma-Aldrich)] for 1 h at room temperature. Coverslips were incubated overnight at 4°C with primary antibodies diluted in PBT [PBS, 0.1% (v/v) Triton X-100 and 1% (w/v) BSA]. The next day, coverslips were washed twice for 5 min in PBT, incubated with secondary antibodies diluted in PBT for 2 h at room temperature, and then washed twice with PBT and once with PBS. Coverslips were mounted on SuperFrost Microscope Slides (VWR) using VECTASHIELD Mounting Medium containing DAPI (Vector Laboratories). Phenotypes were scored in a masked manner by at least two people independently. Images were acquired using a Zeiss Axiovert epifluorescence microscope equipped with µManager software (https://micro-manager.org/). Fiji (Schindelin et al., 2012) was used to generate maximum-intensity projections, which were adjusted for contrast and brightness, and assembled using Photoshop.

### Western blots

Cells were centrifuged, resuspended in PBS, and an equal volume of 2× Laemmli buffer was added. Samples were then boiled for 10 min and stored at −20°C. Proteins were separated by SDS-PAGE and then transferred onto PVDF membranes (Immobilon-P) at 15 V for 1 h. Membranes were blocked overnight at 4°C in PBS containing 0.1% (v/v) Tween (PBST) with 5% (v/v) dry milk powder. After blocking, membranes were washed once with PBST and then incubated with the appropriate primary antibody diluted in PBST containing 5% (v/v) BSA for 2 h at room temperature. Membranes were washed three times for 5 min in PBST and then incubated with HRP-conjugated secondary antibodies in PBST containing 1% BSA for 1 h at room temperature. After an additional three 5 min washes in PBST, the signals were detected using the ECL West Pico substrate (Thermo Fisher Scientific) and chemiluminescence signals were acquired below saturation levels using a G:BOX Chemi XRQ imaging system (Syngene). The intensity of each signal was calculated by first subtracting the relative background intensity and then by normalizing it to the relative tubulin intensity, using the formula (I_S_−I_SB_)/(I_T_−I_TB_), where I_S_ refers to the intensity value of the signal, I_SB_ the value of the background relative to that signal, I_T_ the intensity value of tubulin relative to the signal and I_TB_ the value of the background relative to tubulin. Uncropped images of Western blots are shown in Figs S6–S9.

### Antibodies

CIT-K phospho-specific antibodies were raised in rabbits against synthetic peptides containing either a phosphorylated serine at position 440 (RSESVVSGLDpSPAKTSSMEKK; S440) or a phosphorylated serine at position 699 (EAEERRHpSLENKVKR, S699). Sera were first eluted through a column containing a non-phosphorylated peptide and then each antibody was affinity purified using the appropriate phospho-peptide. Peptide synthesis, conjugation, rabbit immunizations, serum production and affinity purifications were carried out by Generon, UK.

Other antibodies used in this study and their dilutions for western blotting (WB) and immunofluorescence (IF) analyses were: mouse monoclonal anti α-tubulin (clone DM1A, Sigma-Aldrich, T9026; 1:20,000 for WB, 1:2000 for IF), rabbit polyclonal anti-β-tubulin (Abcam, ab6046; 1:5000 for WB, 1:400 for IF), mouse monoclonal anti-cyclin B1 (clone GNS1, Santa Cruz Biotechnology, sc-245; 1:2000 for WB), mouse monoclonal anti-CIT-K (BD Transduction Laboratories, 611377; 1:1500 for WB, 1:250 for IF), rabbit polyclonal anti-KIF14 (Bethyl Laboratories, A300-233A; 1:2000 for WB), rabbit polyclonal anti-MKLP1 (clone N19, Santa Cruz Biotechnology, sc-867; 1:3000 for WB, 1:500 for IF), rabbit recombinant monoclonal anti-MKLP1 antibody (clone EPR10879, Abcam, ab174304; 1:5000 for WB, 1:800 for IF), mouse monoclonal anti-Aurora B (clone AIM-1, BD Transduction Laboratories, 611082; 1:2000 for WB, 1:100 for IF), mouse monoclonal anti-GST (Abcam, ab92; 1:20,000 for WB) and mouse monoclonal anti-maltose-binding protein (MBP) (New England Biolabs, E8032; 1:10,000 for WB). Peroxidase- and Alexa Fluor-conjugated secondary antibodies were purchased from Jackson Laboratories and Thermo Fisher Scientific, respectively. GFP-Booster Alexa Fluor 488 (ChromoTek) was used in immunofluorescence experiments.

### GFP-Trap affinity purification assays

HeLa cells expressing GFP::CIT-K constructs were transfected with an siRNA targeting the 3′ UTR of *CIT-K* and synchronized (120 min after nocodazole release) as described above. Cells were then collected, washed in PBS, frozen in dry ice and stored at −80°C. Cell pellets were resuspended in 0.5 ml of lysis buffer [20 mM Tris-HCl, 150 mM NaCl, 2 mM MgCl_2_, 1 mM EGTA, 0.1% (v/v) NP-40, 1 mM DTT, 5% (v/v) glycerol, Roche cOmplete protease inhibitors and PhosSTOP protein phosphatase inhibitors] and homogenized using a high-performance disperser (Thermo Fisher Scientific). The homogenate was clarified by centrifugation at 750 ***g*** for 15 min at 4°C and the supernatant was incubated with 40 μl of GFP-Trap magnetic Beads (ChromoTek) for 4 h on a rotating wheel at 4°C. Beads were then washed four times in 1 ml of lysis buffer for 5 min on a rotating wheel at 4°C, transferred to a new tube and washed one more time in 1 ml of PBS. After removing as much liquid as possible, beads were resuspended in 2× Laemmli sample buffer, boiled for 5 min and stored at −20°C. Proteins were analyzed by western blotting and quantified using Fiji (Schindelin et al., 2012). To calculate and compare the levels of captured prey proteins, their intensity values were first normalized to the intensity levels of their relative GFP::CIT-K baits, and then compared to the levels of normalized prey proteins captured by the GFP::CIT-K WT bait. The membranes of the western blots were divided in strips to be probed with different antibodies generated in the same host species. This method was designed to correctly quantify the amount of prey proteins because membrane stripping and the use of membranes from different western blots can create incorrect and misleading results.

### *In vitro* GST pull down assays

DNA fragments coding for CIT-K regions were generated by PCR and cloned into either pDEST15 to express N-terminal GST-tagged polypeptides or pKM596 (Fox et al., 2003) to express N-terminal maltose-binding protein (MBP)-tagged polypeptides *in E. coli*. DNA fragments coding for MKLP1_620–858_ and KIF14_901–1648_ were generated by PCR and cloned into pDEST15 to express N-terminal GST tagged polypeptides in *E. coli*. GST-tagged products were then purified using glutathione sepharose 4B beads (GE Healthcare) according to the manufacturer's instruction. MBP-tagged products were purified on amylose resin (New England Biolabs) according to the manufacturer's instruction. The bacteria pellet was resuspended in lysis buffer (20 mM Tris pH 7.5, 250 mM NaCl, 10% glycerol, 0.2% NP-40, 5 mM EDTA pH 8.0, 5 mM DTT, and a cocktail of Roche cOmplete protease inhibitors), sonicated, incubated for 20 min at 4°C, and then centrifuged. Equal volume of lysis buffer with 4% Triton X-100 was then added to the supernatant, which was then loaded on amylose resin beads (New England Bioscience). Beads were washed five times with washing buffer [50 mM Tris-HCl pH 7.5, 250 mM NaCl, 0.2% (v/v) NP-40, 5 mM DTT] and eluted with 20 mM maltose.

For GST pull downs, MBP::CC1 fragments purified and eluted from beads were mixed with 25 μl of glutathione sepharose beads containing purified GST-tagged KIF14_901–1648_ or GST::MKLP1_620–856_ proteins. Samples were incubated in 300 μl of NET-N+ buffer (50 mM Tris-HCl, pH 7.4, 150 mM NaCl, 5 mM EDTA, 0.2% NP-40, and a cocktail of Roche cOmplete protease inhibitors) for 60 min at 4°C on a rotating wheel and then washed five times with 500 μl of wash buffer (50 mM Tris-HCl, pH 7.4, 250 mM NaCl, 5 mM EDTA, 0.2% NP-40, and a cocktail of cOmplete protease inhibitors), followed by centrifugation at 500 ***g*** for 1 min. Beads were resuspended in 25 μl of Laemmli SDS-PAGE sample buffer and analyzed by western blotting. Protein levels were quantified using Fiji (Schindelin et al., 2012).

### Computational and statistical analysis

We used AlphaFold 3 (Abramson et al., 2024) to predict the structure of the longest and only CIT-K isoform that contains S699 (UniProt ID O14578-4). Prism 10 (GraphPad) and Excel (Microsoft) were used for statistical analyses and to prepare graphs.

## Supplementary Material



10.1242/joces.264556_sup1 Supplementary information
