# Supplementary material for: Regulation of Citron kinase by CDK1 and Aurora B regulates midbody formation and stability
Source: J Cell Sci. 2026 May 22;139(10):jcs264556. doi: 10.1242/jcs.264556 (PMC13282566; doi:10.1242/jcs.264556)
Supplement: Supplementary information [file joces-139-264556-s1.pdf]

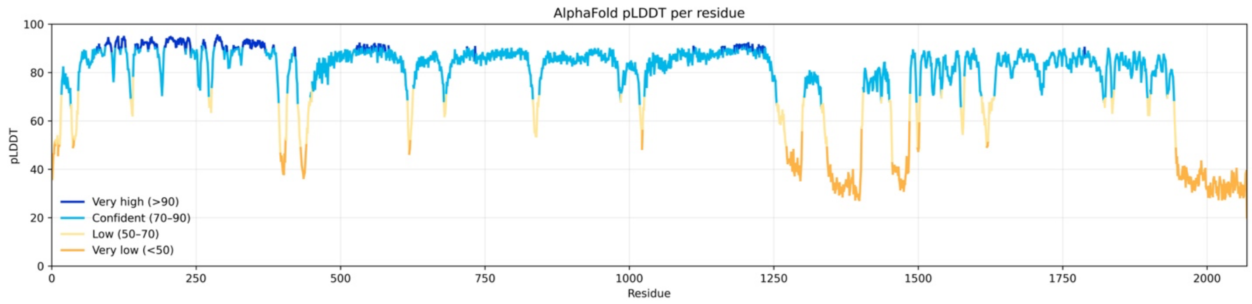

Fig. S1. **Per-residue measure of local confidence of the AlphaFold CIT-K structural model**. Graph illustrating the profile of the AlphaFold predicted Local Distance Difference Test (pLDDT) per residue of the CIT-K structural model shown in Fig. 1E. The color-coding of confidence levels is shown in the bottom left corner.

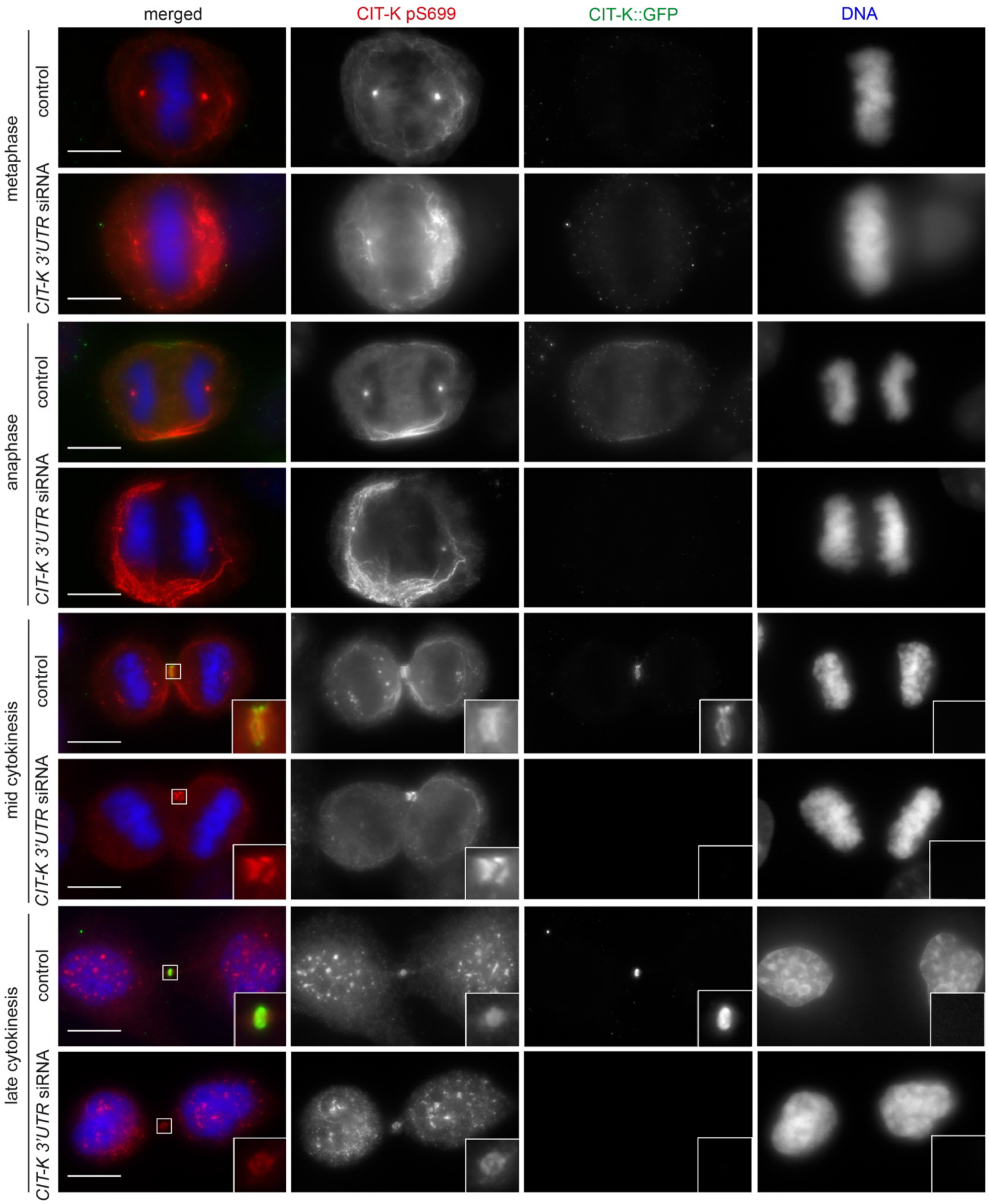

Fig. S2. The CIT-K pS699 antibody does not detect a specific signal in immunofluorescence experiments. HeLa Kyoto cells stably expressing GFP-tagged CIT-K were treated with siRNAs directed against either a random sequence (control) or the coding region of *CIT-K* and after 48 hours were fixed and stained to detect CIT-K::GFP (green), CIT-K pS699 (red) and tubulin (blue). The shape and thickness of microtubule bundles at the intercellular bridge were used as criteria to stage telophase cells. Insets show a 3X magnification of the midbody. Bars, 10 µm.

Journal of Cell Science • Supplementary information

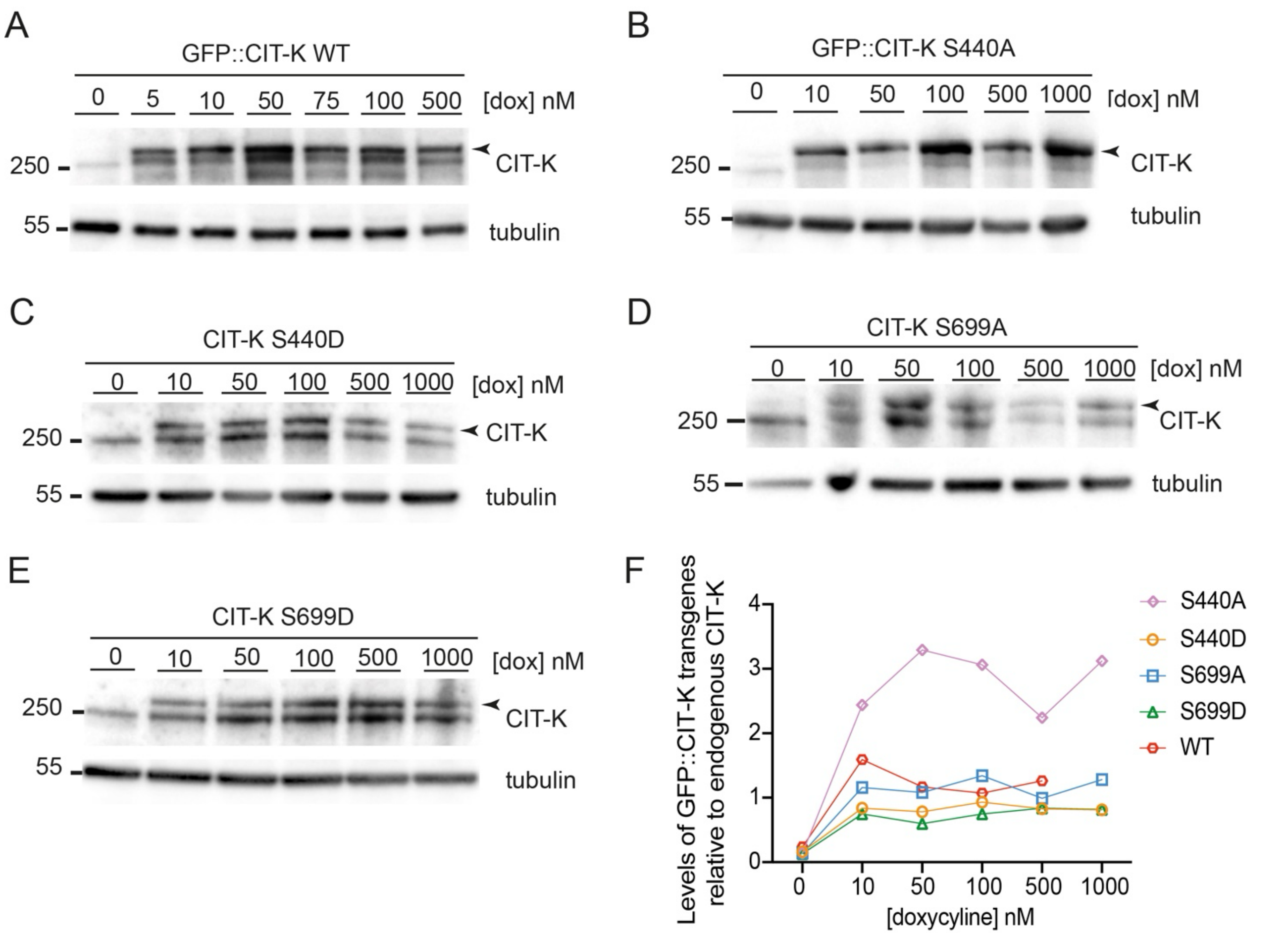

Fig. S3. **Analysis of the level of expression of doxycycline-inducible GFP::CIT-K transgenes**. (A-E) Clones of cell lines stably expressing doxycycline-inducible phospho-dead and phosphomimetic S440 and S699 GFP::CIT-K transgenes were incubated with increasing concentrations (10-1000 nM) of doxycycline (dox). Proteins were extracted and analyzed by Western blot to detect GFP::CIT-K transgenes (indicated by arrowheads), endogenous untagged CIT-K, and tubulin as loading control. The numbers on the left indicate the sizes in kDa of the molecular mass marker. (F) Graph showing the quantification of the protein levels of GFP::CIT-K transgenes, normalized to tubulin and relative to the endogenous CIT-K.

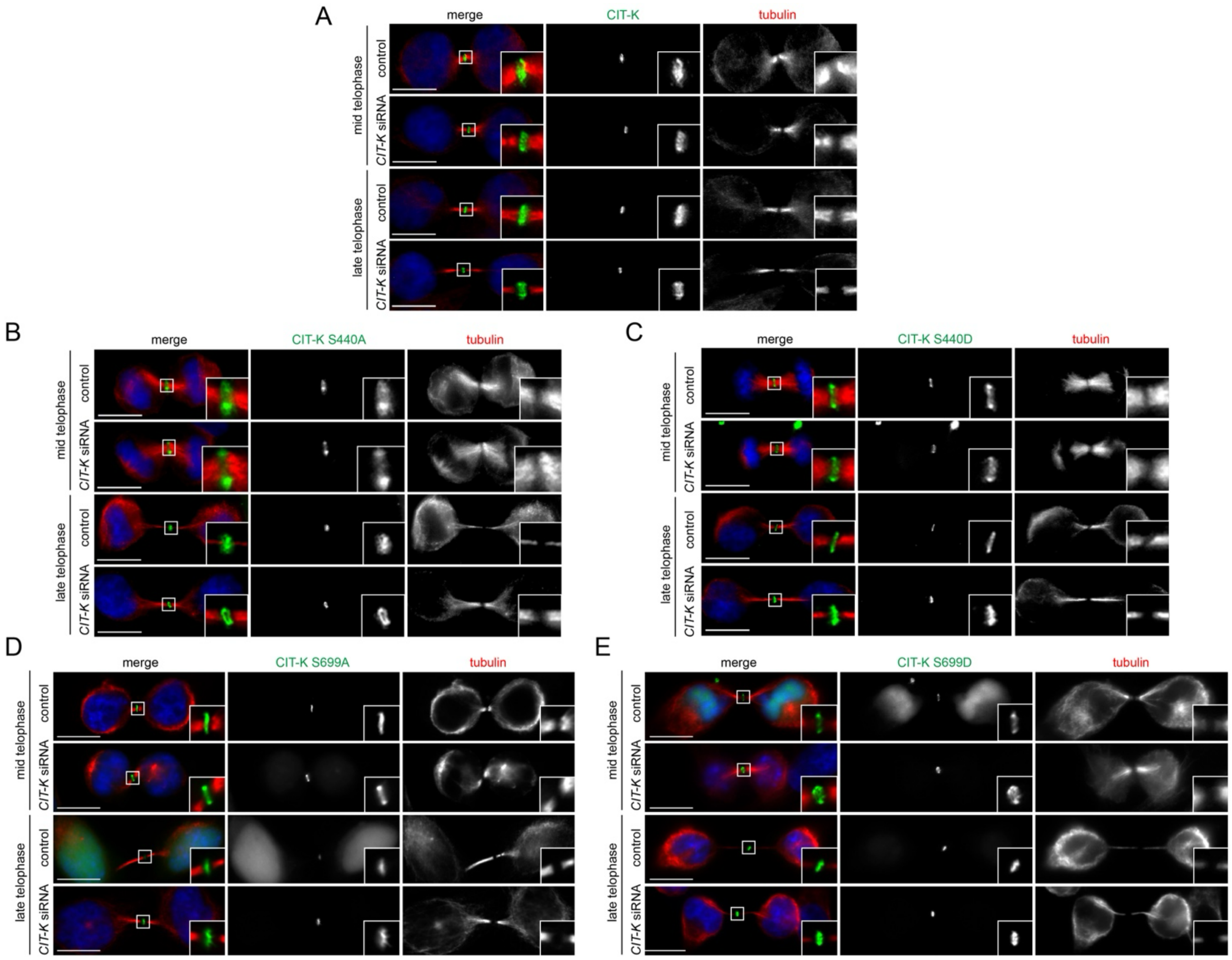

Fig. S4. **Distribution of S440 and S699 phospho-mutant GFP::CIT-K proteins during cytokinesis**. (A-E) HeLa cells expressing wild-type (A) and phospho mutant (B-E) GFP::CIT-K constructs were treated with siRNAs directed against either a random sequence (control) or the 3'UTR of *CIT-K* and after 48 hours were fixed and stained to detect GFP::CIT-K (green), tubulin (red) and DNA (blue). The shape and thickness of microtubule bundles at the intercellular bridge were used as criteria to stage cells. Bars, 10 µm.

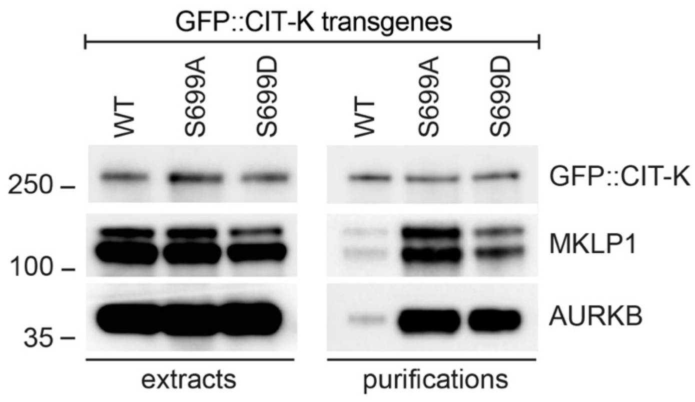

Fig. S5. WT and S699 phospho mutants bind MKLP1/KIF23 and AURKB. HeLa cells stably expressing the indicated GFP-tagged CIT-K transgenes were treated with an siRNA directed against the 3'UTR of *CIT-K*. During RNAi incubation, cells were synchronized by thymidine/nocodazole block and then collected 90 minutes after nocodazole release. Protein extracts were used in a GFP pull-down assay and then analyzed by Western blot to detect the proteins indicated at the right. The numbers on the left indicate the sizes in kDa of the molecular mass marker.

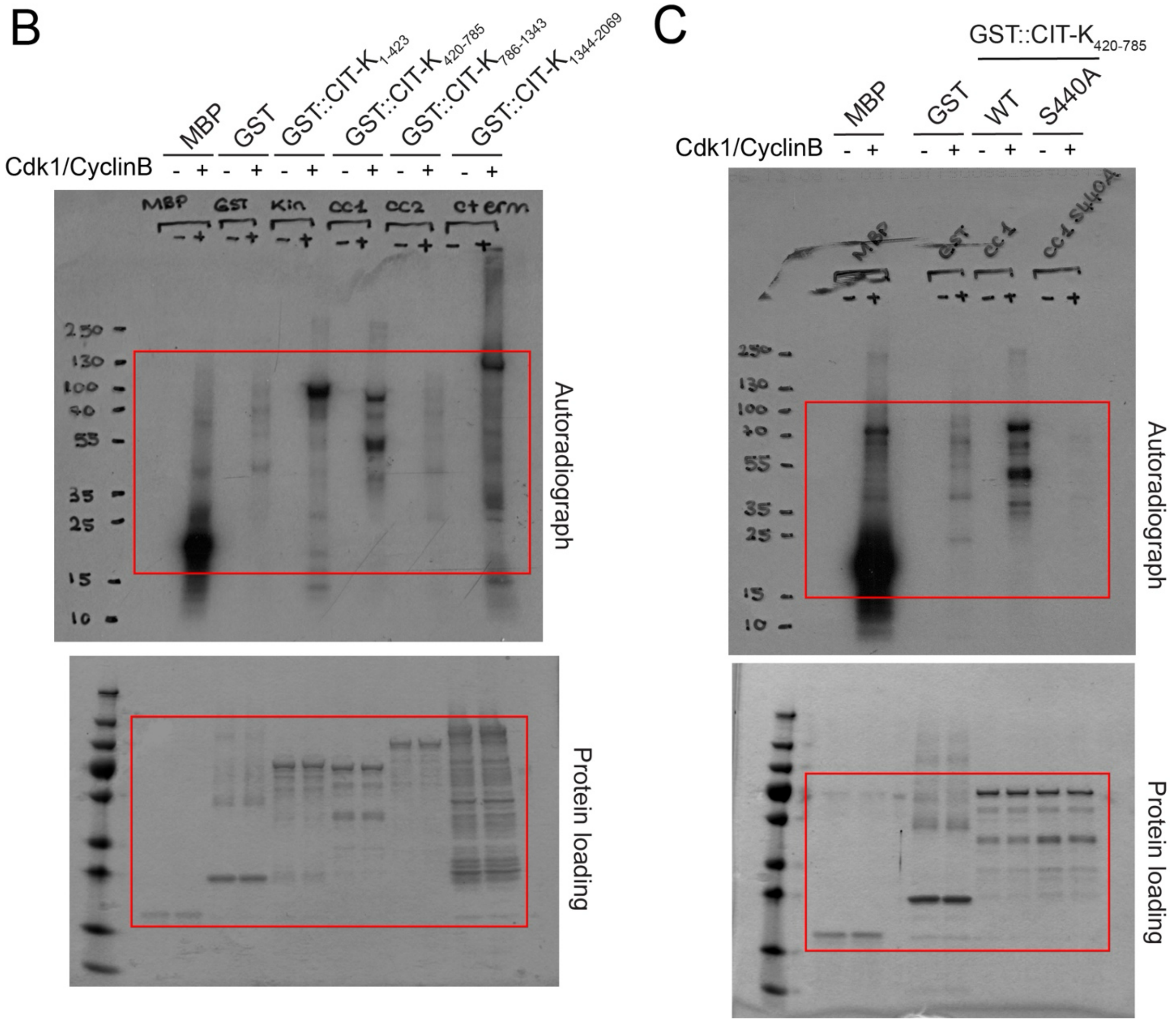

Fig. S6. Uncropped images of the autoradiographs and protein staining of the kinase assays shown in Figure 1. The cropped sections shown in Figure 1 are marked by red rectangles.

Journal of Cell Science • Supplementary information

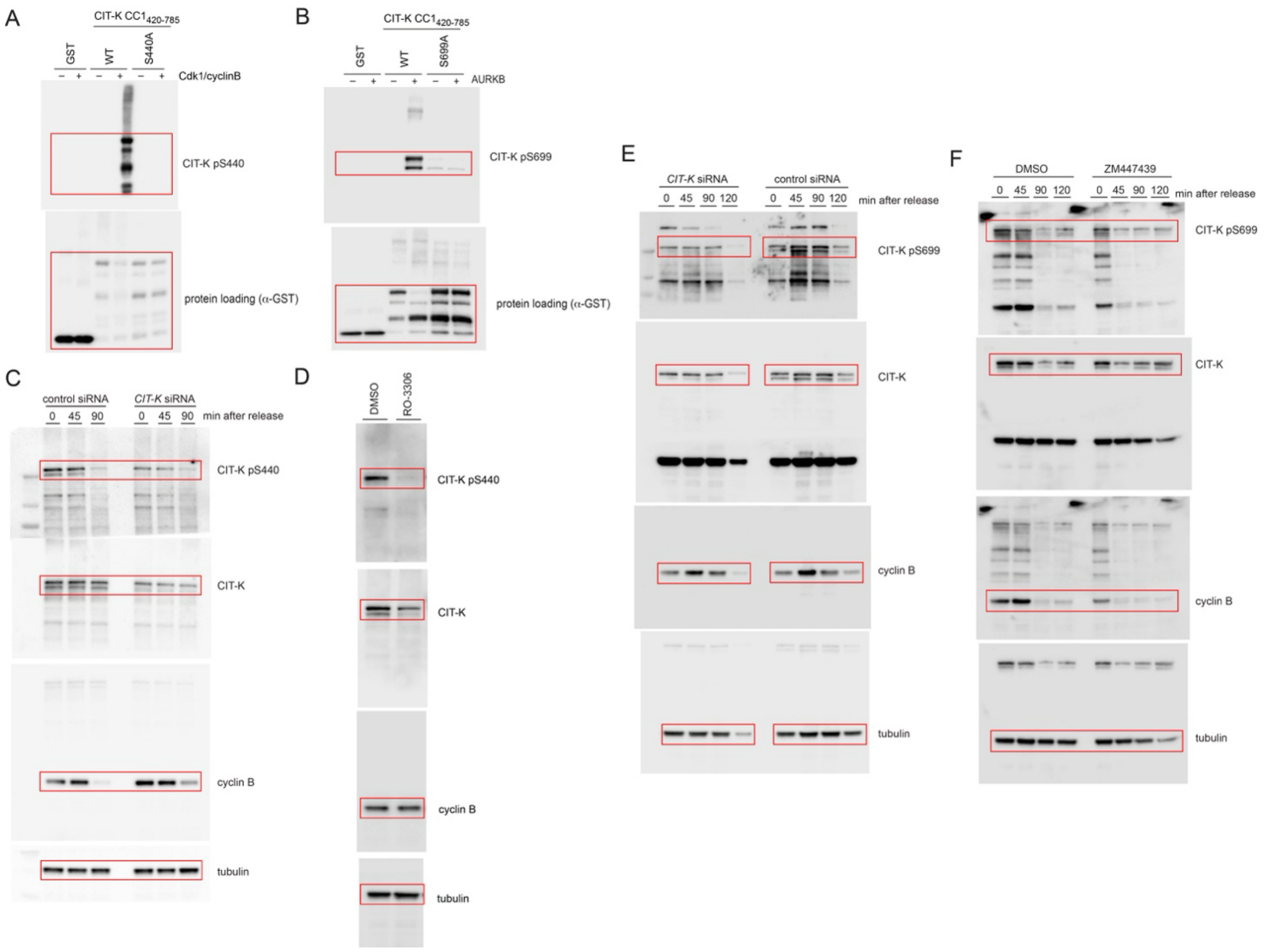

Fig. S7. Uncropped images of the Western blots shown in Figure 2. The cropped sections shown in Figure 2 are marked by red rectangles.

Journal of Cell Science • Supplementary information

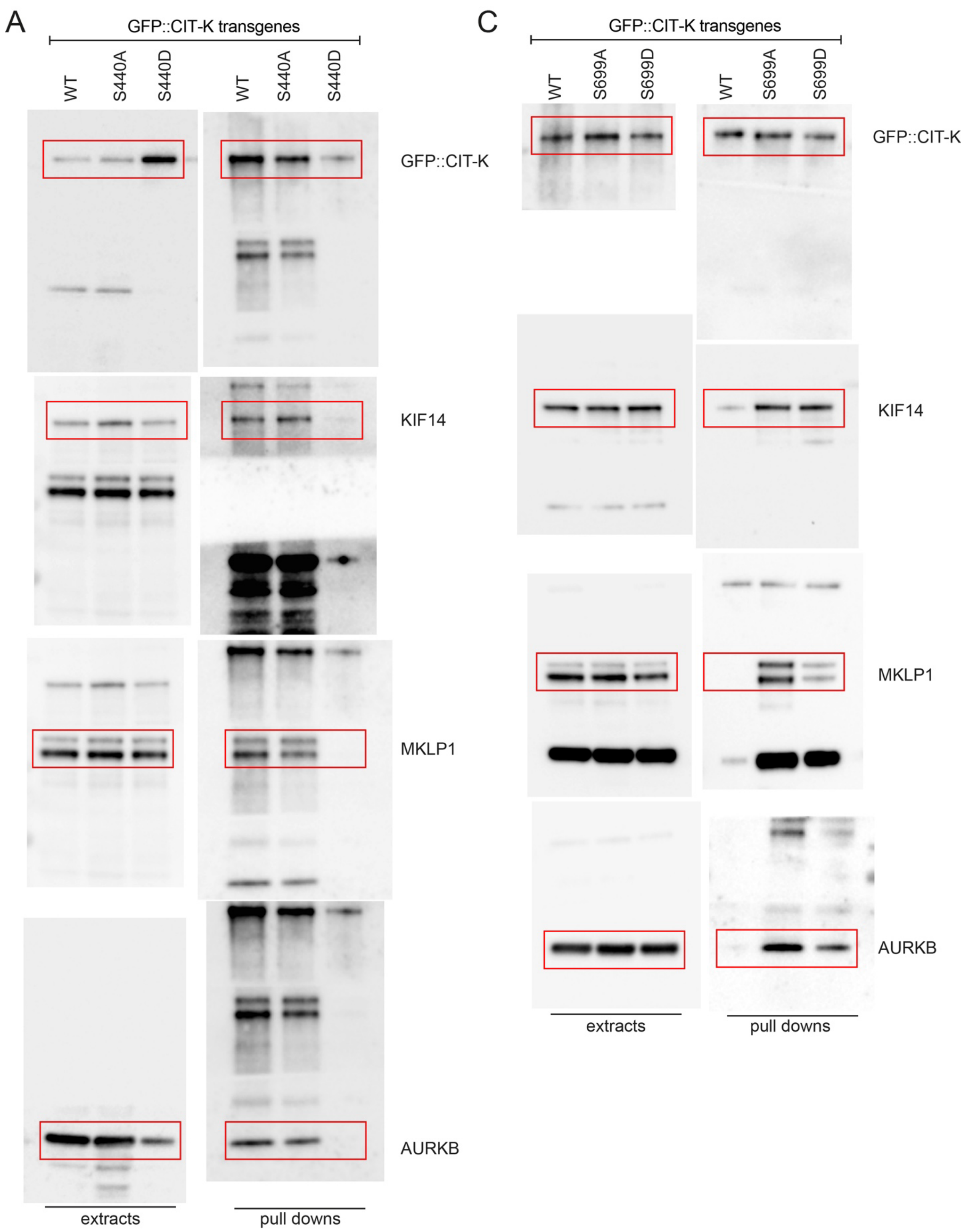

Fig. S8. Uncropped images of the Western blots shown in Figure 5. The cropped sections shown in Figure 5 are marked by red rectangles. The membranes of the Western blots were divided in strips to be probed with different antibodies generated in the same host species. This method was designed to correctly quantify the amount of prey proteins because membrane stripping and the use of membranes from different Western blots can create incorrect and misleading results.

Journal of Cell Science • Supplementary information

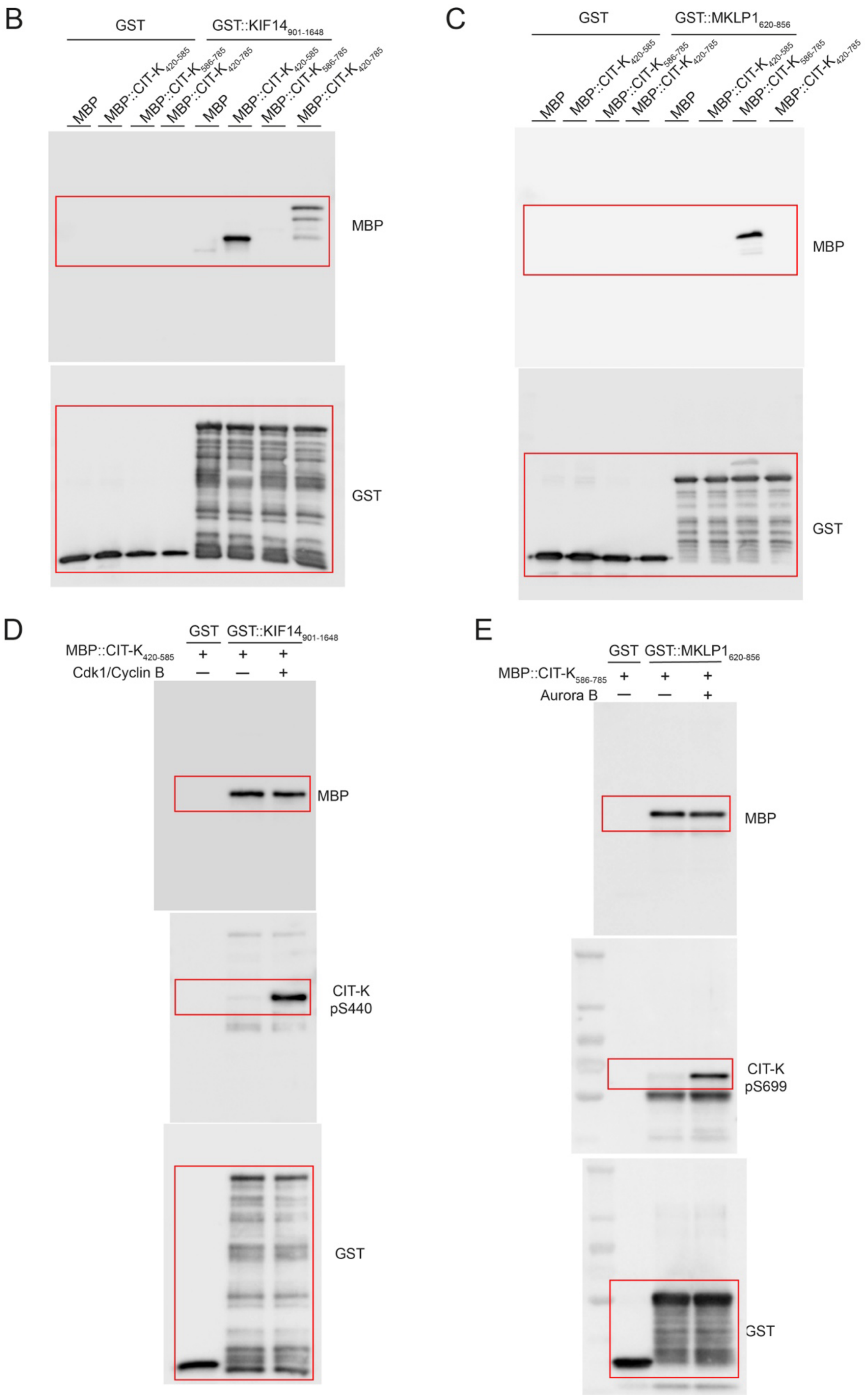

Fig. S9. Uncropped images of the Western blots shown in Figure 6. The cropped sections shown in Figure 6 are marked by red rectangles.

Journal of Cell Science • Supplementary information
