# Supplementary material for: Regulation of Citron kinase by CDK1 and Aurora B regulates midbody formation and stability
Source: J Cell Sci. 2026 May 22;139(10):jcs264556. doi: 10.1242/jcs.264556 (PMC13282566; doi:10.1242/jcs.264556)
Supplement: Supplementary file 1 [file joces-139-264556_review_history.pdf]

Regulation of Citron kinase by CDK1 and Aurora B regulates midbody formation and stability

Luisa Capalbo, Ella Halcrow, Zuni Bassi and Pier Paolo D'Avino
DOI: 10.1242/jcs.264556

Editor: Renata Basto

Review timeline

Original submission:	3 November 2025
Editorial decision:	10 December 2025
First revision received:	29 January 2026
Editorial decision:	2 March 2026
Second revision received:	18 March 2026
Accepted:	7 April 2026

Original submission

First decision letter

MS ID#: jcs.264556

MS TITLE: Coordinated regulation of Citron kinase by CDK1 and Aurora B regulates midbody formation and stability

AUTHORS: Luisa Capalbo; Ella Halcrow; Zuni Bassi; Pier Paolo D'Avino

ARTICLE TYPE: Research Article

Dear Pier Paolo,

We have now reached a decision on the above manuscript.

As you will see, the reviewers raise a number of substantial criticisms that prevent me from accepting the paper at this stage. They suggest, however, that a revised version might prove acceptable, if you can address their concerns. If you think that you can deal satisfactorily with the criticisms on revision, I would be pleased to see a revised manuscript. We would then return it to the reviewers.

Reviewer 1

Advance summary and potential significance to field

The manuscript by D'Avino and colleagues presents a technically rigorous and conceptually novel study dissecting the coordinated regulation of Citron kinase (CIT-K) by CDK1 and Aurora B during cytokinesis, with a focus on two distinct phosphorylation sites, S440 and S699. The work successfully addresses a critical gap in understanding kinase cross-talk at the midbody and offers mechanistic insight into the temporal regulation of CIT-K function, midbody formation, and inheritance, with implications for both cell cycle research and cancer therapeutics. The multi-faceted experimental approach—including cell synchronization, HeLa RNAi rescue, phospho-specific antibodies, live cell imaging, and in vitro biochemistry—ensures robust interrogation of the research questions.

© 2026. Published by The Company of Biologists under the terms of the Creative Commons Attribution License (https://creativecommons.org/licenses/by/4.0/).

Comments for the author

While the study is technically sound and presents compelling findings, several important areas require attention to meet the comprehensive review expectations of the Journal of Cell Science. First, there must be clear documentation that all HeLa-based cell lines used were rigorously authenticated by STR profiling, as misidentified cell lines remain a significant issue in the field. Sample sizes for quantification ($n \geq 3$ biological replicates; 500-1000 cells/condition) are appropriate, but inclusion of power analysis or justification for the sample size would add strength. Quantitative analyses of phenotypes were blind-scored; reporting measures of inter-rater reliability or agreement (e.g., Cohen's kappa) and clarifying the use of randomization in allocation of experimental groups would further enhance rigor. Statistical reporting should also clarify the examination of variance homogeneity and whether multiple hypothesis testing corrections have been systematically applied alongside the use of ANOVA and post-hoc comparisons.

To promote reproducibility, the authors should commit to depositing the plasmids and cell lines used in publicly accessible repositories and clarify whether raw microscopy images and Western blot densitometry data will be made available for independent validation. For antibody validation, especially for phospho-specific reagents, additional controls are needed: inclusion of phosphatase treatment, phospho-peptide competition experiments, and ideally independent mass spectrometric verification of phosphorylation at S440 and S699. The siRNA studies should employ, where feasible, at least two independent oligos per target, or else explicitly demonstrate effective rescue with siRNA-resistant constructs to control for off-target effects.

Regarding biochemical and interaction data, major quantification changes are needed for the co-immunoprecipitation (co-IP) results. Pulldown of CIT-K partners in both wild-type and mutant states must be normalized to both the amount of CIT-K captured and partner input, with data from all biological replicates plotted individually (e.g., as dot plots or box plots), and the normalization and statistical methods concisely described in the figure legends and Methods section. Mechanistically, claims of direct effects on partner binding require further substantiation: as in vitro S440 phosphorylation does not diminish KIF14 binding but S440D reduces co-IP in cells, an on-bead phosphorylation experiment (GFP-Trap isolated wild-type, S440A, and S440D) with recombinant CDK1 and subsequent GST-KIF14 pulldown would clarify if phosphorylation is sufficient to reduce interaction, or whether indirect, potentially allosteric or architectural, effects explain the observed changes. Likewise, conclusions regarding Aurora B binding must account for the substantial spatial separation between the S440/S699 sites and the CNH domain: reduction in cellular co-IP could easily arise from midbody disorganization. The authors should combine acute kinase inhibition (with RO-3306 or ZM447439) with cellular co-IP and immunofluorescence to further dissect localization versus direct affinity effects.

A further concern is that all key functional data derive from GFP-tagged inducible constructs. It is essential for the authors to validate at least the most striking phenotypes (multinucleation, aberrant midbodies, MBR accumulation) using either untagged rescue constructs or, ideally, endogenous CRISPR/Cas9-edited S440A/S440D alleles to confirm physiological relevance and exclude dominant-negative effects of overexpression.

The biological impact of midbody remnant (MBR) accumulation in S440 mutant-expressing cells is intriguing, particularly given reports of MBRs acting as signaling entities or drivers of tumorigenesis. The authors are encouraged to further elaborate on the fate, functional state, and possible intercellular signaling roles of these MBRs, and explicitly discuss the clinical/translational potential of their findings, particularly as Aurora B kinase inhibitors advance in cancer therapy.

Further, the manuscript would benefit from considering evolutionary conservation (noting that S440/S699 motifs are vertebrate-specific), reporting on tissue or cell type specificity of these modifications, and commenting on their potential roles across different organisms. Supplementary figures and methods must be complete and transparent; all protocols and raw data necessary for replication should be provided. For claims based on AlphaFold2 modeling, the authors should report confidence scores (pLDDT) for relevant regions and clarify that structural conclusions require experimental validation.

© 2026. Published by The Company of Biologists under the terms of the Creative Commons Attribution License (https://creativecommons.org/licenses/by/4.0/).

Key recommended text changes include adjusting the Abstract to clarify kinase-site assignments: "We show that CDK1 phosphorylates S440, and Aurora kinases (predominantly Aurora B) phosphorylate S699 within or adjacent to the CIT-K coiled-coil domain." The Results and Methods should precisely describe normalization of co-IP data and the limitations of inferring direct binding effects from cellular co-IP; the Discussion should expand on mechanistic ambiguity in light of midbody organization and domain topology.
In sum, this manuscript provides an important and original contribution to the mechanistic understanding of midbody assembly and cytokinesis, and, with the above revisions—addressing methodological rigor, antibody validation, biochemical quantitation, mechanistic clarity, and translational relevance—will meet the comprehensive standards of Journal of Cell Science and serve as a valuable resource for the field.

The reviewer is an academic cell biologist specializing in cytokinesis and division regulation with no relevant competing interests

Reviewer 2

Advance summary and potential significance to field

This manuscript describes a characterization of two phosphorylation sites on Citron kinase, one phosphorylated by Cdk1 (S440), the other by Aurora B (S699). The evidence that these kinases phosphorylate these sites is compelling, based on both in vitro phosphorylation and in vivo analysis using phospho-specific antibodies that are well validated for western analysis. However, the functional analysis of the role of phosphorylation of these sites is not particularly compelling. Furthermore, there are several examples of major inconsistencies between figures, which really undermines the already modest advance that is claimed.

Comments for the author

Major comments [Please request additional experiments only if they are essential for supporting the conclusions; authors should be encouraged to highlight any claims that are preliminary or speculative, or to discuss any pitfalls or alternative interpretations in a 'Limitations' section]

1) Throughout the manuscript, including the title, abstract and main text, the authors indicate that the phosphoregulation is "coordinated",that there is "cross-talk", or there is a "cross regulatory mechanism". These terms imply that one phosphorylation event impacts the other, but there is no data provided to support that finding. Thus, these terms should be removed. A discussion of the general existence of crosstalk also appears in the introduction, which is also unnecessary.

2) While these studies are conducted in Hela cells which are known to require citron kinase. However, this general requirement for cytokinesis is not supported by mouse genetic analysis in which citron kinase is primarily required in the brain (PMID 11086988). This important piece of background information should be included.

3) The following contradictory sentences appear on pages 6 and 7 respectively.
"These results indicated that the antibodies specifically recognize CIT-K phosphorylated at S440 and S699."
"Notably, the pS440 signal to the midbody core and arms did not colocalize with CIT-K::GFP and did not always disappear after CIT-K RNAi (Fig. 3), suggesting that it might not be specific for CIT-K. However, we cannot exclude that this signal may represent a relatively small, but particularly stable population of CIT-K pS440."
"Unfortunately, the CIT-K pS669 antibody failed to detect any specific signal that was reduced or eliminated after CIT-K RNAi, indicating that this phospho-antibody is not suitable for immunofluorescence experiments (Supplementary Fig. S1)."

Given that the CIT-k siRNA fully depletes the CIT-k signal at the bridge, it is highly unlikely that the residual signal with the phospho-antibody is detecting a stable population. Indeed, while these

© 2026. Published by The Company of Biologists under the terms of the Creative Commons Attribution License (https://creativecommons.org/licenses/by/4.0/).

antibodies have utility for western blotting where reactive species can be size separated, they are not useful for IMF either due to non-specificity (fig 3) or non reactivity (fig S1). Thus these figures are not informative and should not be included as they do not provide any new, reliable information.

4) Functional analysis 1 - figure 4
The strongest effect on cytokinesis is observed with CIT-K699A. However, this is not dominant (no effect in the absence of siRNA), and yet the degree of multinucleation is far higher than depletion of CitK itself.
Furthermore, the results shown in 4C are qualitatively different from those shown in Figure S3 which indicates that all the mutant proteins localize normally. It is hard to know what underlies these differences.

5) Functional analysis 2 - figure 5
This figure is fundamentally flawed. In this experiment, a key control experiment is repeated twice with diametrically different results. In fig 5 A and C, CitK is depleted by RNAi and reconstituted with wild-type CitK. In A, GFP CITK WT efficiently pulls down Kif14, MKLP1 and AURKB, whereas in C only a small amount of Kif14 is recovered, and MKLP1 and AURKB are undetectable. This lack of reproducibility calls into question that results with the phosphomutants. Given that S440D variant generates large, odd looking midbodies, it is possible that its negligible recovery in 5A is due to it being pelleted after lysis.

5) Functional analysis 3 - figure 6
"We then tested whether phosphorylation at S440 or S699 affected the binding of these CIT-K fragments to KIF14 or MKLP1. S440 phosphorylation did not appear to affect the binding of CIT-K420-585 to KIF14 (Fig. 6D), while S699 phosphorylation consistently reduced the association of CIT-K586-785 to MKLP1 in vitro (Fig. 6E)."

Do the authors know whether or not the in vitro phosphorylation reactions generate stoichiometrically phosphorylated MBP:CIT-K fragments? If not these experiments may not measure what the authors claim they are measuring. Could the S->D mutants be used instead, obviating that concern (though a phosphomimetic is not perfect either). The effect of phosphorylation of S699 on MKLP1 binding is not convincing.

Does alphafold predict high confidence interactions between CIT-K and Kif14 and/or MKLP1?

Minor comments

Abstract:
"We show that CDK1 and AURKB phosphorylate two serine residues, S440 and S699, located adjacent to or within CIT-K coiled coil domain."

The abstract should clarify that Cdk1 phosphorylates S440 and AurB phosphorylates S699

Fig 4A Y-axis is misspelled
Fig 4D how is the correct assembly of a midbody assessed in the samples that are represented in this graph?

<u>Reviewer 3</u>

Advance summary and potential significance to field

This paper follows on from previous papers from the same lab investigating the signalling that occurs upstream of citron kinase during cytokinesis in mammalian cells, with particular focus on the kinases, cdk1 and aurora-B.
The authors develop novel phospho-specific antibodies that recognise the modified protein at specific sites (one recognised by each kinase in the C-terminus of citron kinase) and demonstrate that phosphorylation can happen at these sites in vitro using P32 autorads. They show that the

© 2026. Published by The Company of Biologists under the terms of the Creative Commons Attribution License (https://creativecommons.org/licenses/by/4.0/).

PTMS can affect the interaction of citron kinase with certain late mitotic partners, map the regions of interaction to either Kif14 or MKLP1 to distinct domains and relate this to cytokinesis failure and differences in how the midbody remnants are treated as the cells divide.
In my view the paper is too preliminary at this point for publication in JCS. Importantly it lacks certain controls and lacks evidence of multiple repeats and valid statistical analyses that are needed for scientific rigor.
While I have some general comments about the organization (some intro and discussion in the results section etc), I think at this stage it is best to focus on the data presented in the figures and to highlight the limitations that have led to the decision to reject it on the basis of it being too preliminary.

Figure 1.
The legend title says this is about cdk1 and aurora-B kinase phosphorylation in vitro. I only see cdk1 experiments.
1B: blots need to be included showing cyclin B and cdk1 are present in the + lanes; why is MBP showing such high 32P incorporation?
1B and C: the autorads need to be quantitated and N=X and stats presented.
1D: S440 makes sense for a cdk1 site as it is proline directed. By contrast S699 appears to be a non-canonical site for aurora-B. Logo consensus motifs to accompany this motif alignment in different species would be a good addition.
1E: more labels are needed ON the figure - residue numbers, which motif is which etc so that it can be related back to the stick model above it, and later to domain mapping..

Figure 2.
Similar comments as given for Figure 1 - show the kinases added in the blots. Add outlines to the blots so that each experiment can be clearly seen as one. Add kD above marker lane rather than in legend.
In this figure the engineered phospho-specific anitbodies are used in place of the 32P to demonstrate phosphorylation. As these are new reagents, we need to see them used on a whole cell extract +/_ the relevant inhibitor, to demonstrate specificity and back up data that follow in Figure 3.
Are the graphs first normalised to the loading control and then expressed relative to time 0? N=? Stats?+; Label middle panel in B.
2E: can you relate the timings to mitotic phase - a graph of % in each stage at each time point here (and timings on Figure 3 to cross-reference) would be helpful. Were there any apoptotic cells? These should be counted too.
As you go on to do the rescue with CIT-K GFP it is important to show the ectopically expressed form isn't affected by the siRNA (blot).
Organisation- move graph of control alongside the blot and put the siRNA blot underneath. All panels should have their own lettering for clarity.
Quantification of all bands is needed throughout the figure as has been done in (D).

Figure 3:
As noted in figure 2, add timings to the gallery for cross reference to Figure 2. I would call these stages: metaphase, anaphase, telophase, cytokinesis and abscission.
Why is there no signal from the ectopic GFP tagged version in the CITK-GFP siRNA panel, when there is in the control siRNA? (also in S1)
This gallery needs a control +/- cdk1 inhibition to show that the IIF signal is specific.
Figure S1 (accompanies Figure 3).
The assessment criterion for staging is said to be microtubules, but microtubules are not shown here, they are in Figure 3. Staging is fine, but I would call them as noted above.
Also need +/- ZM to show specificity/ lack of specficity the antibody.

Figure S2:
Not sure we need all the dox concentrations to be shown. Just one concentration either side of what was used would suffice. Remove the clone numbers and use simply the construct name to avoid confusion. Label the endogenous and tagged versions on the blot.
S440A seems to be more highly expressed - is endogenous still present in this blot? Is it usurping the endogenous? / affecting its stability?

© 2026. Published by The Company of Biologists under the terms of the Creative Commons Attribution License (https://creativecommons.org/licenses/by/4.0/).

Figure S3:
Text accompanying this figure suggests that S440 mutants can't form proper rings. This I can't tell from the wide field images. Z stacks are needed.
Q: Why is the S440D phenotype "more penetrant" than the S440A if A is the one with higher expression in the blot (Figure S2) and seems to reduce endogenous expression? Would it not be the other way around?

Figure 4:
4A: Quantitation of binucleates is important here in a distinct category from multinucleates, as the latter can form for a variety of reasons, not just cytokinesis failure.
A rise from 4 to 12% after siRNA in control is a rather modest phenotype, though I see this rises further (20%) with S699A expression in place of WT.
Why might S699A cause so much more multinucleation? I'm a little confused as I see both S440 mutants seem to rescue, as does S669D, but S669A does not do so completely.
The persistence of midbody remnants is more convincing.

Figure 5.
The scales on the graphs are very different which is a bit misleading and there are no stats.
5C: why is the WT not pulling down Kif14, MKLP1 and AurkB? It is in 5A and I don't see the difference experimentally. C may have been siRNA'd but I thought this was looking at pull down associated with the transgenic protein not the endogenous?

Figure 6:
This shows some domain mapping of interactions with Kif14 or MKLP1. I have similar comments to Figure 2 and 3. The experimental concept is good, but it needs to show the kinases added, carry out further repeats and include statistical analyses.

First revision

Author response to reviewers' comments

Reviewer 1
The manuscript by D'Avino and colleagues presents a technically rigorous and conceptually novel study dissecting the coordinated regulation of Citron kinase (CIT-K) by CDK1 and Aurora B during cytokinesis, with a focus on two distinct phosphorylation sites, S440 and S699. The work successfully addresses a critical gap in understanding kinase cross-talk at the midbody and offers mechanistic insight into the temporal regulation of CIT-K function, midbody formation, and inheritance, with implications for both cell cycle research and cancer therapeutics. The multi-faceted experimental approach—including cell synchronization, HeLa RNAi rescue, phospho-specific antibodies, live cell imaging, and in vitro biochemistry—ensures robust interrogation of the research questions. We are pleased that the Reviewer found our study rigorous, informative and novel. The Reviewer made a few comments, which we found to be thoughtful and helpful. Our point-by-point responses to the Reviewer’s comments are below.

While the study is technically sound and presents compelling findings, several important areas require attention to meet the comprehensive review expectations of the Journal of Cell Science. First, there must be clear documentation that all HeLa-based cell lines used were rigorously authenticated by STR profiling, as misidentified cell lines remain a significant issue in the field. We understand this concern. We used two HeLa cell lines, HeLa Kyoto and HeLa T-REx. The HeLa Kyoto cell line has been fully characterized (PMID: 23550136) and was tested and authenticated in other previous studies (PMID: 22724069, PMID: 23716662, PMID: 27009191, PMID: 27784789, PMID: 31586073). The HeLa T-REx cell line is commercially available and was purchased from ThermoFisher Scientific (see Materials and Methods).

Sample sizes for quantification (n ≥ 3 biological replicates; 500-1000 cells/condition) are

© 2026. Published by The Company of Biologists under the terms of the Creative Commons Attribution License (https://creativecommons.org/licenses/by/4.0/).

appropriate, but inclusion of power analysis or justification for the sample size would add strength. Quantitative analyses of phenotypes were blind-scored; reporting measures of inter-rater reliability or agreement (e.g., Cohen's kappa) and clarifying the use of randomization in allocation of experimental groups would further enhance rigor. Statistical reporting should also clarify the examination of variance homogeneity and whether multiple hypothesis testing corrections have been systematically applied alongside the use of ANOVA and post-hoc comparisons.
We appreciate the Reviewer's suggestion. We use specific and objective criteria for phenotypic analysis, including the structure of the intercellular bridge, the assembly of the central spindle microtubule arrays, and the correct distribution of midbody markers. This method significantly reduces inter- and intra-rater variability and therefore we do not think that it is necessary to report "*measures of inter-rater reliability or agreement*". Out statistical analyses are described in the figure legends and Materials and Methods.

To promote reproducibility, the authors should commit to depositing the plasmids and cell lines used in publicly accessible repositories and clarify whether raw microscopy images and Western blot densitometry data will be made available for independent validation.
We have always made our reagents available to the scientific community after publication, and this time will be no different. Our raw microscopy and Western blot densitometry data will be available upon request. Please note that we provided uncropped images of all our autoradiographs and Western blots in the Supplementary material.

For antibody validation, especially for phospho-specific reagents, additional controls are needed: inclusion of phosphatase treatment, phospho-peptide competition experiments, and ideally independent mass spectrometric verification of phosphorylation at S440 and S699.
We mentioned at the beginning of the Results section that S440 and S699 phosphorylations were initially identified by proteomic analyses of CIT-K affinity purifications from cells and *in vitro* phosphorylation experiments (page 5 and Fig. 1A). In addition, we present a series of experiments in the manuscript to extensively validate the phospho-antibodies:

a) We employed *in vitro* phosphorylation assays to confirm that the antibodies specifically recognize the CIT-K fragment phosphorylated *in vitro* by CDK1 and AURKB, and not if the S440 and S669 residues were mutated to alanine (Fig 2A and 2B);
b) We used siRNA depletion to confirm that the phospho-antibodies specifically detect CIT-K in cell extracts (Fig. 2C and 2E);
c) We show that the signals detected by these phospho-antibodies are reduced if cells are treated with CDK1 and AURKB inhibitors (Fig. 2D and 2F);
d) We show that the pS440 CIT-K signal decreases after siRNA treatment in immunofluorescence experiments (Fig. 3).

We think that these results confirm that our antibodies are specific, selective, and reproducible.

Phospho- and non phospho-peptide competitions were used for the initial screening and selection of the sera from two different immunized animals. Furthermore, as described in Materials and Methods, "*sera were first eluted through a column containing a non-phosphorylated peptide and then each antibody was affinity purified using the appropriate phospho-peptide*".

As for the comment on the inclusion of phosphatase treatment, we have not yet identified the phosphatase(s) responsible for S440 and S699 dephosphorylation. We know that their de-phosphorylation is inhibited after treatment with okadaic acid (data not shown), suggesting that PP1 and/or PP2A phosphatases might be involved. However, these are preliminary data that need further investigation and are not relevant to this study. If the Reviewer meant to suggest treatment of cell extracts with a general phosphatase (e.g., λ phosphatase), this would only prove that the antibodies recognize a phosphorylated substrate without any indication about their specificity for CIT-K S440 and S699. By contrast, our experiments in Fig. 2 convincingly confirm that the antibodies specifically recognize CIT-K phosphorylated at S440 and S699.

The siRNA studies should employ, where feasible, at least two independent oligos per target, or else explicitly demonstrate effective rescue with siRNA-resistant constructs to control for off- target effects.
As described in Materials and Methods, "*these siRNAs have been previously validated for*

© 2026. Published by The Company of Biologists under the terms of the Creative Commons Attribution License (https://creativecommons.org/licenses/by/4.0/).

specificity and efficacy (McKenzie et al., 2016)".

Regarding biochemical and interaction data, major quantification changes are needed for the co- immunoprecipitation (co-IP) results. Pulldown of CIT-K partners in both wild-type and mutant states must be normalized to both the amount of CIT-K captured and partner input, with data from all biological replicates plotted individually (e.g., as dot plots or box plots), and the normalization and statistical methods concisely described in the figure legends and Methods section.

We think that there might have been some confusion about the quantification of affinity purified prey proteins. The aim of these experiments was to assess if the introduction of phospho-mutations affected CIT-K association with its partners. The graphs in Fig. 5B and 5D show the normalized values of the prey proteins relative to the levels of those purified by the GFP::CIT-K wild type (WT) bait. To calculate and compare the levels of captured prey proteins, their intensity values were first normalized to the intensity levels of their relative GFP-CIT-K baits, and then compared to the levels of normalized prey proteins captured by the GFP::CIT-K wild type (WT) bait. We apologize if this was not clear and have explained our calculations in more detail in both figure legends and Materials and Methods.

We disagree that the values of the pull downs of CIT-K partners should be normalized to "*both the amount of CIT-K captured and partner input*" because the preys are not from co-transfected plasmids, but endogenous proteins. The amounts of purified preys have to be normalized only to the amounts of the specific baits.

We think that bar charts showing mean, SEM and individual data values are appropriate and useful to visualize one variable with a limited number of distinct values. However, following the Reviewer's comment and according to the recommendations of the journal, we have changed the graph style to dot plots and included data from an additional experimental replicate in the revised Fig. 5 to further validate our results.

Mechanistically, claims of direct effects on partner binding require further substantiation: as in vitro S440 phosphorylation does not diminish KIF14 binding but S440D reduces co-IP in cells, an on-bead phosphorylation experiment (GFP-Trap isolated wild-type, S440A, and S440D) with recombinant CDK1 and subsequent GST-KIF14 pulldown would clarify if phosphorylation is sufficient to reduce interaction, or whether indirect, potentially allosteric or architectural, effects explain the observed changes. Likewise, conclusions regarding Aurora B binding must account for the substantial spatial separation between the S440/S699 sites and the CNH domain: reduction in cellular co-IP could easily arise from midbody disorganization. The authors should combine acute kinase inhibition (with RO-3306 or ZM447439) with cellular co-IP and immunofluorescence to further dissect localization versus direct affinity effects.

Our IP assays indicate that S440 phosphorylation inhibits CIT-K association with AURKB, KIF14 and MKLP1 (Fig. 5). Our *in vitro* pull down assays indicate that GST:: KIF14901-1648 specifically pulled down the CIT-K420-585 fragment containing S440, but this binding was not altered by S440 phosphorylation (Fig. 6). As mentioned in our Discussion, together "*these results indicate that phosphorylation at S440 (and S669) in cells must have a more widespread effect on CIT-K's ability to interact with its partners than just local inhibition*". We think that the use of *in vitro* phosphorylated GFP-tagged CIT- K proteins instead of bacterially purified CIT-K fragments would not further clarify the nature of the specific effect(s) of S440 phosphorylation because CDK1 phosphorylates other two serines, S400 and S1982 (Fig. 1A). Therefore, this alternative method would very likely lead to less informative and potentially misleading results.

We sincerely do not understand the Reviewer's comment about the potential effect of the spatial separation between the S440/S699 sites and the CNH domain on Aurora B binding. Aurora B is the enzymatic component of the chromosomal passenger complex (CPC). We previously reported that the CPC strongly binds to the CNH domain of CIT-K (McKenzie et al, PMID: 27009191) and, considering the size of the CPC, we think that any speculation about the spatial separation between Aurora B to S440/S699 would be arbitrary and inaccurate. Furthermore, we can exclude the possibility that reduction of Aurora B purification might be caused by midbody disorganization because our affinity purification methodology has been optimized to specifically avoid the precipitation of midbodies and large protein complexes. As described in

© 2026. Published by The Company of Biologists under the terms of the Creative Commons Attribution License (https://creativecommons.org/licenses/by/4.0/).

Materials and Methods, we centrifuge cell extracts only once at low speed (750 *g)* precisely to avoid this possibility. We then use magnetic beads for the purification. Notably, this methodology worked very successfully for many affinity purifications, including the characterization of the midbody interactome (PMID: 31586073). We have now changed the labelling “pull downs” with “purifications” in Fig.5A and 5C to avoid possible confusion.

Finally, we found in previous experiments that treating cells with CDK1 and AURKB inhibitors led to inconsistent, unreliable and confusing results about protein localization and interactions, most likely because these inhibitors affected the phosphorylation status of a large number of mitotic proteins with pleiotropic and knock-on effects. By contrast, experiments with phospho mutants produce more reliable and specific results.

A further concern is that all key functional data derive from GFP-tagged inducible constructs. It is essential for the authors to validate at least the most striking phenotypes (multinucleation, aberrant midbodies, MBR accumulation) using either untagged rescue constructs or, ideally, endogenous CRISPR/Cas9-edited S440A/S440D alleles to confirm physiological relevance and exclude dominant-negative effects of overexpression.
The results presented in this study and in others published previously have indicated that CIT-K tagged with GFP or other tags can rescue depletion of the endogenous protein, indicating that GFP tagging does not affect the functions of CIT-K (PMID: 27009191, PMID: 27895316).

Using CRISPR gene editing to generate mutant proteins may seem a valid option in theory, but in practice it is usually impossible to generate these cell lines because mutant variants of essential proteins are very likely to be unfunctional and/or toxic.

The biological impact of midbody remnant (MBR) accumulation in S440 mutant-expressing cells is intriguing, particularly given reports of MBRs acting as signaling entities or drivers of tumorigenesis. The authors are encouraged to further elaborate on the fate, functional state, and possible intercellular signaling roles of these MBRs, and explicitly discuss the clinical/translational potential of their findings, particularly as Aurora B kinase inhibitors advance in cancer therapy.
The recent findings indicating that MBRs act as short-range and long-term intercellular signaling organelles that can also transmit transformative properties are extremely interesting and exciting. We did mention these studies in our Introduction and provide relative citations. However, we do not investigate the roles of CIT-K S440 and S699 phosphorylation in post-mitotic midbody functions and tumorigenesis in this study. Therefore, we think that it would not be appropriate to further speculate on post-mitotic MBR functions that would not be supported by our results.

Further, the manuscript would benefit from considering evolutionary conservation (noting that S440/S699 motifs are vertebrate-specific), reporting on tissue or cell type specificity of these modifications, and commenting on their potential roles across different organisms.
We show that S440 and S699 are evolutionary conserved in vertebrates (Fig. 1D), but we have not investigated if their phosphorylation is conserved in other species. We also have not analyzed if these phosphorylations are tissue-specific. Although these questions are interesting, we think that speculating about evolutionary conservation and potential roles in different species and tissues without any supporting results would be premature.

Supplementary figures and methods must be complete and transparent; all protocols and raw data necessary for replication should be provided. For claims based on AlphaFold2 modeling, the authors should report confidence scores (pLDDT) for relevant regions and clarify that structural conclusions require experimental validation.
We are surprised by this comment because our supplementary figures are transparent. We think that our experimental procedures are described in detail, but have included additional information to describe Western blot quantification and analysis in more detail. We also provided uncropped images of all Western blots (Supplementary Figures S5 to S8) and included the confidence scores for the AlphaFold prediction analysis in Supplementary Figure S1.

Key recommended text changes include adjusting the Abstract to clarify kinase-site assignments:

© 2026. Published by The Company of Biologists under the terms of the Creative Commons Attribution License (https://creativecommons.org/licenses/by/4.0/).

"We show that CDK1 phosphorylates S440, and Aurora kinases (predominantly Aurora B) phosphorylate S699 within or adjacent to the CIT-K coiled-coil domain." The Results and Methods should precisely describe normalization of co-IP data and the limitations of inferring direct binding effects from cellular co-IP; the Discussion should expand on mechanistic ambiguity in light of midbody organization and domain topology.
We have modified the Abstract to specifically indicate that S440 is phosphorylated by CDK1 and S699 by Aurora B. The quantifications of affinity purification data were described in figure legends; we have now included them also in the Materials and Methods. We think that offering any speculation about mechanistic ambiguity would be inappropriate without supporting results.

In sum, this manuscript provides an important and original contribution to the mechanistic understanding of midbody assembly and cytokinesis, and, with the above revisions—addressing methodological rigor, antibody validation, biochemical quantitation, mechanistic clarity, and translational relevance—will meet the comprehensive standards of Journal of Cell Science and serve as a valuable resource for the field.
We are pleased that the Reviewer found our study original and informative. We think that our replies and modifications have addressed the Reviewer's comments.

Reviewer 2
This manuscript describes a characterization of two phosphorylation sites on Citron kinase, one phosphorylated by Cdk1 (S440), the other by Aurora B (S699). The evidence that these kinases phosphorylate these sites is compelling, based on both in vitro phosphorylation and in vivo analysis using phospho-specific antibodies that are well validated for western analysis. However, the functional analysis of the role of phosphorylation of these sites is not particularly compelling. Furthermore, there are several examples of major inconsistencies between figures, which really undermines the already modest advance that is claimed.
We are pleased that the Reviewer found our analysis of CIT-K phosphorylation valid. They expressed some comments about the functional analysis of these phosphorylation events and inconsistencies between figures. Our point-by-point responses to these comments are below.

Major comments

1) Throughout the manuscript, including the title, abstract and main text, the authors indicate that the phosphoregulation is "coordinated",that there is "cross-talk", or there is a "cross regulatory mechanism". These terms imply that one phosphorylation event impacts the other, but there is no data provided to support that finding. Thus, these terms should be removed. A discussion of the general existence of crosstalk also appears in the introduction, which is also unnecessary.
We did not say, suggest or speculate that there could be interdependency between CDK1 and Aurora B phosphorylation events. We understand that the adjective "*coordinated*" might be potentially misinterpreted, as this regulation could be considered as a combined effort. We have therefore removed "coordinated" throughout the manuscript.
We disagree that the use of the cross-talk term is inappropriate because this is a cross-regulative mechanism between mitotic kinases, which is also supported by our previous study (McKenzie et al., 2016, *Cross-regulation between Aurora B and Citron kinase controls midbody architecture in cytokinesis*, PMID: 27009191).

2) While these studies are conducted in Hela cells which are known to require citron kinase. However, this general requirement for cytokinesis is not supported by mouse genetic analysis in which citron kinase is primarily required in the brain (PMID 11086988). This important piece of background information should be included.
This comment is not completely accurate. There is evidence that CIT-K and other midbody proteins are particularly important for brain development in rodents and humans, but CIT-K is also required for cytokinesis in mouse spermatogenic precursors (PMID: 12432070) and in a large variety of human cell lines (PMID: 27895316). Furthermore, there is evidence that some CIT-K functions in brain development might be different between mice and humans (PMID: 39316437). Finally, we do not investigate the role of CIT-K phosphorylation in brain development and therefore it would not be relevant to discuss this topic in our manuscript. We cited articles and reviews that discuss this aspect of CIT-K function in the Introduction.

© 2026. Published by The Company of Biologists under the terms of the Creative Commons Attribution License (https://creativecommons.org/licenses/by/4.0/).

3) The following contradictory sentences appear on pages 6 and 7 respectively.
"These results indicated that the antibodies specifically recognize CIT-K phosphorylated at S440 and S699."
"Notably, the pS440 signal to the midbody core and arms did not colocalize with CIT-K::GFP and did not always disappear after CIT-K RNAi (Fig. 3), suggesting that it might not be specific for CIT-K. However, we cannot exclude that this signal may represent a relatively small, but particularly stable population of CIT-K pS440."
"Unfortunately, the CIT-K pS669 antibody failed to detect any specific signal that was reduced or eliminated after CIT-K RNAi, indicating that this phospho-antibody is not suitable for immunofluorescence experiments (Supplementary Fig. S1)."
Given that the CIT-k siRNA fully depletes the CIT-k signal at the bridge, it is highly unlikely that the residual signal with the phospho-antibody is detecting a stable population. Indeed, while these antibodies have utility for western blotting where reactive species can be size separated, they are not useful for IMF either due to non-specificity (fig 3) or non reactivity (fig S1). Thus these figures are not informative and should not be included as they do not provide any new, reliable information.
These sentences, which are taken from different contexts, are not contradictory.

The first sentence stating that "*the antibodies specifically recognize CIT-K phosphorylated at S440 and S699*" comes after the description of the Western blot analyses of cell extracts from control CIT- K RNAi depleted cells. These results convincingly demonstrate that the phosphoantibodies are specific.

The second sentence follows the description of immunofluorescence experiments that revealed the detection of pS440 signals "*to the midbody core and arms did not colocalize with CIT-K::GFP and did not always disappear after CIT-K RNAi*". Importantly, this discrepancy was observed only in mid and late cytokinesis cells. The pS440 signals in metaphase, anaphase and early cytokinesis cells colocalize with GFP::CIT-K and are reduced or absent after CIT-K RNAi (Fig. 3), supporting the specificity of the pS440 antibody. We have now clarified this in the revised manuscript. We think that the possibility that the pS440 signal at the midbody may represent a relatively small, but particularly stable population of CIT-K pS440, cannot be excluded because antibody staining is much more sensitive than GFP fluorescence.

The third sentence, indicating that "*the CIT-K pS669 antibody failed to detect any specific signal that was reduced or eliminated after CIT-K RNAi, indicating that this phospho-antibody is not suitable for immunofluorescence experiments)*" does not contradict the first sentence. It is not unusual that antibodies may work well for just one type of essay.

To conclude, we present a series of experiments in the manuscript to extensively validate the phospho-antibodies:

a) We employed *in vitro* phosphorylation assays to confirm that the antibodies specifically recognize the CIT-K fragment phosphorylated *in vitro* by CDK1 and AURKB, and not if the S440 and S669 residues were mutated to alanine (Fig 2A and 2B);
b) We used siRNA depletion to confirm that the phospho-antibodies specifically detect CIT-K in cell extracts (Fig. 2C and 2E);
c) We show that the signals detected by these phospho-antibodies are reduced if cells are treated with CDK1 and AURKB inhibitors (Fig. 2D and 2F);
d) We show that the pS440 CIT-K signal decreases after siRNA treatment in immunofluorescence experiments (Fig. 3).

We think that these results confirm that our antibodies are specific, selective, and reproducible.

We think that Figures 3 and S1 are valid and informative and thus there is no reason for excluding them.

4) Functional analysis 1 - figure 4
The strongest effect on cytokinesis is observed with CIT-K699A. However, this is not dominant (no effect in the absence of siRNA), and yet the degree of multinucleation is far higher than depletion of CitK itself.
The point of this comment is not clear. Cells expressing the CIT-K S699A mutant have a basal frequency of multinucleated cells higher than cells without any transgene (empty) after

© 2026. Published by The Company of Biologists under the terms of the Creative Commons Attribution License (https://creativecommons.org/licenses/by/4.0/).

control siRNA treatment. Consistently, the frequency of multinucleation of CIT-K S699 cells after CIT-K RNAi is also higher than that observed in cells without transgene (empty) (Fig. 4A). Notably, the fold increases of multinucleation after CIT-K RNAi in the two cell lines are very similar, 3.21 for no transgene (empty) cells and 2.66 for S699A cells. We attached below a copy of the Data Table for this graph.

Table format: Grouped		Group A				Group B			
		control				3'UTR siRNA			
		A:Y1	A:Y2	A:Y3	A:Y4	B:Y1	B:Y2	B:Y3	B:Y4
1	empty	3.92	2.86	4.09	4.17	10.06	11.70	13.05	13.48
2	WT	3.27	3.63	3.50	6.95	3.50	8.25	6.01	8.78
3	S440A	4.69	3.60	3.50	3.88	3.55	3.09	3.98	4.09
4	S400D	5.58	4.95	4.61	5.83	4.23	3.19	5.83	4.79
5	S699A	5.87	8.38	8.86	6.63	23.40	20.69	17.84	16.50
6	S699D	5.59	6.19	8.10	8.63	7.23	8.53	8.08	9.83

Furthermore, the results shown in 4C are qualitatively different from those shown in Figure S3 which indicates that all the mutant proteins localize normally. It is hard to know what underlies these differences.

We disagree with the Reviewer’s comment that the results shown in Figure S3 and Figure 4 are “*qualitatively different*”. The experiments shown in these figures are complementary and both consistently support the evidence that expression of S440 mutants affects midbody assembly and organization.

Figure S3 illustrates that all mutant proteins are recruited to the midbody and that in some cases S440A and S440D mutant CIT-K proteins “*failed to assemble in the ring-like structure typical of CIT- K and showed excessive accumulation to the midbody in very late cytokinesis, just before abscission, a phenotype that was more penetrant for the S440D mutant (Supplementary Fig. S3B-C)*”. Fig 4C extends this analysis by showing representative images of cells with incorrectly assembled midbodies co-stained with the midbody components and CIT-K interactors, Aurora B, KIF14 and MKLP1/KIF23. These results confirmed that these structures represented entire midbodies and not just aggregates of the GFP::CIT-K mutant proteins. Moreover, they showed that the midbody distribution of these three CIT-K interactors was altered in cells expressing S440A and S440D mutants.

5) Functional analysis 2 - figure 5

This figure is fundamentally flawed. In this experiment, a key control experiment is repeated twice with diametrically different results. In fig 5 A and C, CitK is depleted by RNAi and reconstituted with wild-type CitK. In A, GFP CITK WT efficiently pulls down Kif14, MKLP1 and AURKB, whereas in C only a small amount of Kif14 is recovered, and MKLP1 and AURKB are undetectable. This lack of reproducibility calls into question that results with the phosphomutants. Given that S440D variant generates large, odd looking midbodies, it is possible that its negligible recovery in 5A is due to it being pelleted after lysis.

This comment is incorrect, Figure 5 is not flawed. The results of the affinity purifications using CIT- K WT in the two figures are not “*diametrically different*”. In both cases, the WT protein associated with KIF14, MKLP1 and Aurora B. The signals of MKLP1 and Aurora B are weaker in Fig. 5C compared to Fig. 5A simply because the exposure was shorter. As indicated by the graph in Fig. 5D, the S669A mutation enhanced the association of CIT-K with MKLP1 and Aurora B very strongly, 10 times more than WT. This is a consistent result, also supported by our previously published experiment using a different cell line expressing the S699A mutant (see Figs. 7e and S5 in McKenzie et al., *Cross- regulation between Aurora B and Citron kinase controls midbody architecture in cytokinesis*, PMID: 27009191). We have mentioned this in the revised text.

We can exclude the possibility that reduction of purification of CIT-K partners might be caused by midbody disorganization because our affinity purification methodology has been optimized to specifically avoid the precipitation of midbodies and large protein complexes. As described in Materials and Methods, we centrifuge cell extracts only once at low speed (750 *g)* precisely to avoid this possibility. We then use magnetic beads for the purification. Notably, this methodology worked very successfully for many affinity purifications, including the characterization of the midbody interactome (PMID: 31586073). We have now changed the labelling “pull downs” with

© 2026. Published by The Company of Biologists under the terms of the Creative Commons Attribution License (https://creativecommons.org/licenses/by/4.0/).

"purifications" in Fig.5A and 5C to avoid possible confusion.

5) Functional analysis 3 - figure 6
"We then tested whether phosphorylation at S440 or S699 affected the binding of these CIT-K fragments to KIF14 or MKLP1. S440 phosphorylation did not appear to affect the binding of CIT-K420-585 to KIF14 (Fig. 6D), while S699 phosphorylation consistently reduced the association of CIT-K586-785 to MKLP1 in vitro (Fig. 6E)."
Do the authors know whether or not the in vitro phosphorylation reactions generate stoichiometrically phosphorylated MBP:CIT-K fragments? If not these experiments may not measure what the authors claim they are measuring. Could the S->D mutants be used instead, obviating that concern (though a phosphomimetic is not perfect either). The effect of phosphorylation of S699 on MKLP1 binding is not convincing.
It is not possible to accurately measure the level of phosphorylated substrates in these *in vitro* phosphorylation experiments. However, our protocol is designed to reach maximum kinase activity, according to both manufacturer's quality test (attached below) and our own optimization. Furthermore, antibody staining confirmed that phosphorylation occurred in both experiments at high level (Figs 6D and 6E). Therefore, it is highly unlikely that phosphorylation of the fragments had negligible effect on the binding. We performed binding experiments using the phosphomimetic S440D mutant, but the results were no different, indicating that it is not a valid alternative.

CDK1/cyclin B Activity Graph

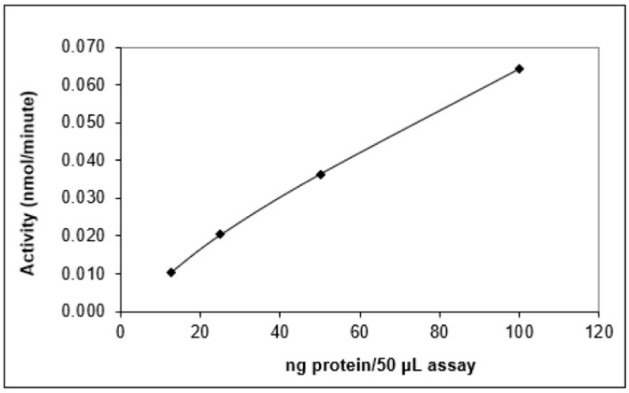

Dilution Buffer:

20 mM Tris (pH 7.5), 0.05% Triton® X–100, 0.1 mg/mL BSA, 1 mM DTT and 10% Glycerol.

Assay Conditions:

CDK1/cyclin B was pre-diluted in enzyme dilution buffer and assayed in 20 mM Tris (pH 7.5), 5 mM $MgCl_2$, 100 µM ATP, 100 µg/mL Histone H1 peptide substrate (PKTPKKAKKL) and trace [^{32}P]-γ-ATP for 10 minutes at 30°C.

Does alphafold predict high confidence interactions between CIT-K and Kif14 and/or MKLP1?
We have not carried out this analysis. These interactions have been experimentally demonstrated in this and previously published studies (PMID: 16431929, PMID: 23444367, PMID: 23716662, PMID: 27009191).

Minor comments
Abstract:
"We show that CDK1 and AURKB phosphorylate two serine residues, S440 and S699, located adjacent to or within CIT-K coiled coil domain."
The abstract should clarify that Cdk1 phosphorylates S440 and AurB phosphorylates S699
We have modified the Abstract accordingly.

Fig 4A Y-axis is misspelled
We apologize for this typo, which has been corrected.

Fig 4D how is the correct assembly of a midbody assessed in the samples that are represented in this graph?

© 2026. Published by The Company of Biologists under the terms of the Creative Commons Attribution License (https://creativecommons.org/licenses/by/4.0/).

Correct midbody assembly is shown in the WT images. We use specific and objective criteria for phenotypic analysis of midbodies, including the structure of the intercellular bridge, the assembly of the central spindle microtubule arrays, and the correct distribution of midbody markers.

Reviewer 3

This paper follows on from previous papers from the same lab investigating the signalling that occurs upstream of citron kinase during cytokinesis in mammalian cells, with particular focus on the kinases, cdk1 and aurora-B.

The authors develop novel phospho-specific antibodies that recognise the modified protein at specific sites (one recognised by each kinase in the C-terminus of citron kinase) and demonstrate that phosphorylation can happen at these sites in vitro using P32 autorads. They show that the PTMS can affect the interaction of citron kinase with certain late mitotic partners, map the regions of interaction to either Kif14 or MKLP1 to distinct domains and relate this to cytokinesis failure

and differences in how the midbody remnants are treated as the cells divide.

In my view the paper is too preliminary at this point for publication in JCS. Importantly it lacks certain controls and lacks evidence of multiple repeats and valid statistical analyses that are needed for scientific rigor.

While I have some general comments about the organization (some intro and discussion in the results section etc), I think at this stage it is best to focus on the data presented in the figures and to highlight the limitations that have led to the decision to reject it on the basis of it being too preliminary.

The Reviewer does not recommend publication of our manuscript because in their opinion it is "*too preliminary*". Unfortunately, the Reviewer did not expand on the specific reasons why our study would be preliminary, but just mentioned the lack of unspecified controls, repeats and statistical analysis.

The Reviewer decided to offer a list of very short and mostly technical comments that we have addressed below.

Figure 1.

The legend title says this is about cdk1 and aurora-B kinase phosphorylation in vitro. I only see cdk1 experiments.

As stated in the paper, *in vitro* phosphorylation of S699 by Aurora B was described in our previous study (McKenzie et al., 2016, *Cross-regulation between Aurora B and Citron kinase controls midbody architecture in cytokinesis*, PMID: 27009191).

1B: blots need to be included showing cyclin B and cdk1 are present in the + lanes; why is MBP showing such high 32P incorporation?

Fig 1B and 1C show the results of *in vitro* phosphorylation experiments. The CDK1/cyclin B complex was included (+) or omitted (-) in the reactions, resulting in extremely clear differences between the two conditions. The successful phosphorylations observed in these experiments automatically confirmed the presence of the kinase and therefore there was no need to carry out a Western blot analysis on radioactive membranes to confirm its presence. The myelin basic protein (MBP) is a widely used positive control for CDK1 phosphorylation.

1B and C: the autorads need to be quantitated and N=X and stats presented.

These qualitatively experiments convincingly and clearly confirm that S440 is directly phosphorylated by CDK1. We obtained almost identical results in two separate experiments.

1D: S440 makes sense for a cdk1 site as it is proline directed. By contrast S699 appears to be a non-canonical site for aurora-B. Logo consensus motifs to accompany this motif alignment in different species would be a good addition.

In our previous study we showed that S699 matched the consensus for Aurora B kinase (see table S2 in McKenzie et al., *Cross-regulation between Aurora B and Citron kinase controls midbody architecture in cytokinesis*, PMID: 27009191).

The evolutionary conservation of this site and sequence alignment in vertebrates is shown in Fig. 1D.

1E: more labels are needed ON the figure - residue numbers, which motif is which etc so that it can be related back to the stick model above it, and later to domain mapping.

We explained in the figure legend that the CIT-K domains in the AlphaFold model have been color- coded as the schematic diagram in Fig. 1A.

© 2026. Published by The Company of Biologists under the terms of the Creative Commons Attribution License (https://creativecommons.org/licenses/by/4.0/).

Figure 2.
Similar comments as given for Figure 1 - show the kinases added in the blots. Add outlines to the blots so that each experiment can be clearly seen as one. Add kD above marker lane rather than in legend.
We explained before why there is no need to carry out a Western blot analysis to show the presence of the kinases. The blots are clearly visible and we prefer not to include kD after the numbers indicating the sizes of the marker bands.
In this figure the engineered phospho-specific anitbodies are used in place of the 32P to demonstrate phosphorylation. As these are new reagents, we need to see them used on a whole cell extract +/_ the relevant inhibitor, to demonstrate specificity and back up data that follow in Figure 3.
The Reviewer must have missed that the experiments with specific kinase inhibitors are shown in
Figs. 2D and 2F.
Are the graphs first normalized to the loading control and then expressed relative to time 0? N=? Stats?+; Label middle panel in B.
We explained in the figure legend that protein levels were normalized to tubulin and relative to the zero time point. We have now included this information also in the Materials and Methods. There is no middle panel in Figure 2B.
2E: can you relate the timings to mitotic phase – a graph of % in each stage at each time point here (and timings on Figure 3 to cross-reference) would be helpful. Were there any apoptotic cells? These should be counted too.
This comment is confusing. Figure 2E shows Western blots of protein extracts from synchronized cells, whereas Figure 3 shows immunofluorescence images of unsynchronized cells. Mitotic exit can be monitored by cyclin B degradation in the Western blots in Fig. 2E.

We used the thymidine-nocodazole block and release procedure for cell synchronization. This method has been widely used for decades by many laboratories, including ours (PMID: 27009191, PMID: 27784789, PMID: 31586073, PMID: 32878943). Apoptosis is generally very low (<5%), which clearly indicates that our synchronization method does not significantly affect cell viability.
As you go on to do the rescue with CIT-K GFP it is important to show the ectopically expressed form isn't affected by the siRNA (blot).
The Reviewer probably missed that these results are shown in Figs 2C and 2D, where the endogenous untagged CIT-K is marked by arrows. These panels show that the slower migrating form corresponding to GFP::CIT-K is not reduced after RNAi.
Organisation- move graph of control alongside the blot and put the siRNA blot underneath. All panels should have their own lettering for clarity.
We think that our current figure organization is clear for readers and there is no need to make any changes. All panels have their own lettering.
Quantification of all bands is needed throughout the figure as has been done in (D).
The quantifications of panels 2C, 2E and 2F are shown in their accompanying graphs.

Figure 3:
As noted in figure 2, add timings to the gallery for cross reference to Figure 2. I would call these stages: metaphase, anaphase, telophase, cytokinesis and abscission.
Figure 3 shows immunofluorescence images of unsynchronized cells - there are no timings to add. We think that the nomenclature suggested by the Reviewer would be inaccurate because telophase cells are in cytokinesis and abscission has not yet occurred in the late cytokinesis cell.
Why is there no signal from the ectopic GFP tagged version in the CITK-GFP siRNA panel, when there is in the control siRNA? (also in S1)
The siRNA used in these experiments depleted both endogenous and GFP-tagged CIT-K because it targets the coding region of CIT-K. We realized that there was an error in the figure legend that has now been corrected.
This gallery needs a control +/- cdk1 inhibition to show that the IIF signal is specific.
The specificity of this antibody for CDK1-phosphorylated S440 is shown in Fig. 2D. The aim of this immunofluorescence experiment is to show the distribution of pS440 CIT-K and verify the antibody

© 2026. Published by The Company of Biologists under the terms of the Creative Commons Attribution License (https://creativecommons.org/licenses/by/4.0/).

specificity for CIT-K. CDK1 inhibition would not determine that the observed signals are specific for pS440 CIT-K. In addition, treating cells with CDK1 and AURKB inhibitors affects the phosphorylation status of a large number of mitotic proteins, leading to very abnormal mitotic figures and inconsistent, unreliable and confusing results about protein localization.

Figure S1 (accompanies Figure 3).
The assessment criterion for staging is said to be microtubules, but microtubules are not shown here, they are in Figure 3. Staging is fine, but I would call them as noted above.
Also need +/- ZM to show specificity/ lack of specficity the antibody.

We stained these cells also for microtubules, but we showed DNA in the figure. We have discussed above the issues relative to the use of the Aurora B inhibitor in immunofluorescence experiments.

Figure S2:
Not sure we need all the dox concentrations to be shown. Just one concentration either side of what was used would suffice. Remove the clone numbers and use simply the construct name to avoid confusion. Label the endogenous and tagged versions on the blot.

We used different doxycycline concentrations to establish the correct concentration to use for each construct in order to obtain expression levels similar to the endogenous untagged CIT-K. We prefer to show the entire experiment without eliminating any information. The clone numbers have been removed as requested by the Reviewer. The bands corresponding to the GFP tagged CIT-K proteins had been marked by arrowheads.

S440A seems to be more highly expressed - is endogenous still present in this blot? Is it usurping the endogenous? / affecting its stability?

The faster migrating band corresponding to endogenous untagged CIT-K is evident in this Western blot. We have no evidence indicating that expression of GFP::CIT-K S440A may affect the expression or stability of the endogenous protein.

Figure S3:
Text accompanying this figure suggests that S440 mutants can't form proper rings. This I can't tell from the wide field images. Z stacks are needed.

We think that the images shown in Figure S3 clearly show that S440A and S440D proteins failed to assemble in the ring-like structure typical of CIT-K and showed excessive accumulation to the midbody in very late cytokinesis. These results are further confirmed by the experiments shown in Fig. 4C. We think that it would be unnecessary and superfluous to show Z stacks for this supplementary figure.

Q: Why is the S440D phenotype "more penetrant" than the S440A if A is the one with higher expression in the blot (Figure S2) and seems to reduce endogenous expression? Would it not be the other way around?

The penetrance of a phenotype is determined by the nature of the mutation and not necessarily by the expression level.

Figure 4:
4A: Quantitation of binucleates is important here in a distinct category from multinucleates, as the latter can form for a variety of reasons, not just cytokinesis failure.

Binucleate cells were of course included in our counting of multinucleate cells. We included all cells containing 2 or more equally sized nuclei. Cells containing micronuclei were of course not included. A rise from 4 to 12% after siRNA in control is a rather modest phenotype, though I see this rises further (20%) with S699A expression in place of WT.

We disagree that a threefold increase in multinucleate cells can be considered “*modest*” and this is
confirmed by statistical analysis (Fig. 4A). Cells expressing the CIT-K S699A mutant have a basal frequency of multinucleate cells higher than cells without any transgene (empty) after control siRNA treatment. Consistently, the frequency of multinucleation of CIT-K S699 cells after CIT-K RNAi is also higher than that observed in cells without transgene (empty) (Fig. 4A). Notably, the fold increases of multinucleation after CIT-K RNAi in the two cell lines are very similar, 3.21 for no transgene (empty) cells and 2.66 for S699A cells. We attached a copy of the Data Table for this graph in the reply to Reviewer 1.

Why might S699A cause so much more multinucleation? I'm a little confused as I see both S440 mutants seem to rescue, as does S669D, but S669A does not do so completely.

© 2026. Published by The Company of Biologists under the terms of the Creative Commons Attribution License (https://creativecommons.org/licenses/by/4.0/).

The persistence of midbody remnants is more convincing.
CITK S699 failed to rescue depletion of endogenous CIT-K, but it did not cause more multinucleation. All the other mutants rescued.

Figure 5.
The scales on the graphs are very different which is a bit misleading and there are no stats.
5C: why is the WT not pulling down Kif14, MKLP1 and AurkB? It is in 5A and I don't see the difference experimentally. C may have been siRNA'd but I thought this was looking at pull down associated with the transgenic protein not the endogenous?
The scales of the two graphs are different because they report data from two different experiments.

The results of the affinity purifications using CIT-K WT in Figures 5A and 5C are not discordant. In both cases, the WT protein associated with KIF14, MKLP1 and Aurora B. The signals of MKLP1 and Aurora B are weaker in Fig. 5C compared to Fig. 5A simply because the exposure was shorter. As indicated by the graph in Fig. 5D, the S669A mutation enhanced the association of CIT-K with MKLP1 and Aurora B very strongly, about 10 times more than WT. This is a consistent result, in agreement with our previously published experiment using a different cell line expressing the S699A mutant (see Figs. 7e and S5 in McKenzie et al., *Cross-regulation between Aurora B and Citron kinase controls midbody architecture in cytokinesis*, PMID: 27009191). We have mentioned this in the revised text.

Figure 6:
This shows some domain mapping of interactions with Kif14 or MKLP1. I have similar comments to Figure 2 and 3. The experimental concept is good, but it needs to show the kinases added, carry out further repeats and include statistical analyses.
The identification and mapping of the domains involved in these interactions were published previously (PMID: 16431929, PMID: 23444367, PMID: 23716662, PMID: 27009191).

We explained before why there is no need to perform a Western blot analysis on radioactive membranes to confirm the presence of the kinases. Furthermore, antibody staining confirmed that phosphorylation occurred in both experiments at high level (Figs 6D and 6E).

The results of the two replicates are consistent and statistical analysis would not be conceptually correct because the graphs show relative values from a limited (n=2) number of samples.

Second decision letter

MS ID#: jcs.264556R1

MS TITLE: Regulation of Citron kinase by CDK1 and Aurora B regulates midbody formation and stability

AUTHORS: Luisa Capalbo; Ella Halcrow; Zuni Bassi; Pier Paolo D'Avino

ARTICLE TYPE: Research Article

Dear Paolo,

Your revised article has been seen by two of the three reviewers that initially reviewed it. Reviewer 3 is happy with the revised version and only requests a few minor clarifications. Reviewer 2 points out to some important discrepancies, namely relate with Figure 5 that we think have to be addressed. Moreover, this reviewer also pointed out that in Fig S7 (and most likely S6), the uncropped blots do not appear to display the same level of contrast/exposure, and some of the lanes don’t align. This may be explained by the fact that you cut/ probed/stripped different membranes and then reassembled them, but it is important to precisely describe this, i.e. how the membranes were processed and then imaged.

© 2026. Published by The Company of Biologists under the terms of the Creative Commons Attribution License (https://creativecommons.org/licenses/by/4.0/).

If you think that you can deal satisfactorily with the criticisms on revision, I would be pleased to see a revised manuscript.

Reviewer 2: SUMMARY OF THE ADVANCE MADE IN THIS PAPER AND ITS POTENTIAL SIGNIFICANCE TO THE FIELD

The updates to my previous comments are presented following the comments from the initial review

This manuscript describes a characterization of two phosphorylation sites on Citron kinase, one phosphorylated by Cdk1 (S440), the other by Aurora B (S699). The evidence that these kinases phosphorylate these sites is compelling, based on both in vitro phosphorylation and in vivo analysis using phospho-specific antibodies that are well validated for western analysis. However, the functional analysis of the role of phosphorylation of these sites is not particularly compelling. Furthermore, there are several examples of major inconsistencies between figures, which really undermines the already modest advance that is claimed.

****
The updates to my previous comments are presented following the comments from the initial review. Such comments are bracketed by *** like this one.

****

****

Many of the issues from the previous reviews were not adequately addressed.

****

1) Throughout the manuscript, including the title, abstract and main text, the authors indicate that the phosphoregulation is "coordinated",that there is "cross-talk", or there is a "cross regulatory mechanism". These terms imply that one phosphorylation event impacts the other, but there is no data provided to support that finding. Thus, these terms should be removed. A discussion of the general existence of crosstalk also appears in the introduction, which is also unnecessary.

****

In the revised version the abstract states "Here, we report the characterization of a novel cross-talk mechanism by which CDK1 and Aurora B (AURKB) kinases regulate the distribution and interactions of Citron kinase (CIT-K)."

Likewise the end of the discussion states,
"Here, we report a novel cross-regulatory mechanism among mitotic kinases important for the formation and stability of the midbody."

No evidence for cross-talk is reported or demonstrated in this manuscript.

****

2) While these studies are conducted in Hela cells which are known to require citron kinase. However, this general requirement for cytokinesis is not supported by mouse genetic analysis in which citron kinase is primarily required in the brain (PMID 11086988). This important piece of background information should be included.

****

The fact that mice lacking citron kinase are viable (and male sterile) shows that Citron kinase is not generally required for cytokinesis in all vertebrate cells. The authors reply states that "Finally, we do not investigate the role of CIT-K phosphorylation in brain development and therefore it would

© 2026. Published by The Company of Biologists under the terms of the Creative Commons Attribution License (https://creativecommons.org/licenses/by/4.0/).

not be relevant to discuss this topic in our manuscript." yet the manuscript states that "Finally, some midbody proteins have been linked to brain development and microcephaly (Siskos et al., 2021)." Why don't the authors want to provide readers with the full biological context?
Likewise, Figure 1D only shows the presence of these sites in vertebrates. Are these sites conserved in invertebrates?

****

3) The following contradictory sentences appear on pages 6 and 7 respectively.
"These results indicated that the antibodies specifically recognize CIT-K phosphorylated at S440 and S699."
"Notably, the pS440 signal to the midbody core and arms did not colocalize with CIT-K::GFP and did not always disappear after CIT-K RNAi (Fig. 3), suggesting that it might not be specific for CIT-K. However, we cannot exclude that this signal may represent a relatively small, but particularly stable population of CIT-K pS440."
"Unfortunately, the CIT-K pS669 antibody failed to detect any specific signal that was reduced or eliminated after CIT-K RNAi, indicating that this phospho-antibody is not suitable for immunofluorescence experiments (Supplementary Fig. S1)."

Given that the CIT-k siRNA fully depletes the CIT-k signal at the bridge, it is highly unlikely that the residual signal with the phospho-antibody is detecting a stable population. Indeed, while these antibodies have utility for western blotting where reactive species can be size separated, they are not useful for IMF either due to non-specificity (fig 3) or non reactivity (fig S1). Thus these figures are not informative and should not be included as they do not provide any new, reliable information.

****

While it is reasonable to conclude that the pS440 shown in Figure 3 "early cytokinesis" does represent phosphorylated CitK as it colocalizes with the tagged protein and is sensitive depletion of CitK by RNAi it is incorrect to conclude that the signal observed in "mid" and "late" cytokinesis represents pS440 CitK as (1) this signal does not colocalize with GFP-CITK and (2) is not subject to depletion by CitK RNAi. The argument for differential detection are unconvincing, the signals in early cytokinesis are comparable, why would this change later during cytokinesis? Bottom line, the phospho S440 antibody fails both tests for specificity in late cytokinesis, so it is not clear why the authors would want to include results that are entirely unconvincing.

****
****
3B) The origin of graph in fig 2F should be set to 0, not 0.4 (as in Figure 2C)
****

4) Functional analysis 1 - figure 4
The strongest effect on cytokinesis is observed with CIT-K699A. However, this is not dominant (no effect in the absence of siRNA), and yet the degree of multinucleation is far higher than depletion of CitK itself.

****
The point I was making is that the inhibitory effect of CIT-K 699A is not observed in the presence of the WT protein, indicating that this is not acting as a dominant negative. Furthermore, the effect of this allele is greater than the loss of CIT-K itself, suggesting it is not a loss of function phenotype, together that indicates that CIT-K is a gain of function interfering allele. The lack of dominance could be that in the presence of the WT, a large fraction of this protein dimerizes with the WT and AurB phosphorylation of either subunit suffices to prevent its from acting dominantly. This is consistent with Fig 7f of McKenzie et al.

In any case, given that both S440D and S699D largely rescue multinucleation that dynamic phosphorylation at these sites are not essential. This key finding should be clearly stated.
****

© 2026. Published by The Company of Biologists under the terms of the Creative Commons Attribution License (https://creativecommons.org/licenses/by/4.0/).

5) Functional analysis 2 - figure 5
This figure is fundamentally flawed. In this experiment, a key control experiment is repeated twice with diametrically different results. In fig 5 A and C, CitK is depleted by RNAi and reconstituted with wild-type CitK. In A, GFP CITK WT efficiently pulls down Kif14, MKLP1 and AURKB, whereas in C only a small amount of Kif14 is recovered, and MKLP1 and AURKB are undetectable. This lack of reproducibility calls into question that results with the phosphomutants. Given that S440D variant generates large, odd looking midbodies, it is possible that its negligible recovery in 5A is due to it being pelleted after lysis.

****
This analysis remains fundamentally flawed. The lack of consistency between Fig 5A and 5C in the WT lanes is not adequately explained by differential exposures. None of the exposures shown in the supplement support this notion.
Note the authors should clarify the images in this supplemental figure. It is apparent, but not-stated that the filters were cut up, probed with different antibodies and reassembled. In the reassembled figure it is not transparent which part of the filters were probed with which antibody.

Secondly, the failure to recover GFP-CITK-S440D in the purifications renders the assessment of its interacting proteins moot. It is a failed experiment. Why publish an experiment that failed due to technical reasons?

Third, in Fig 7fe of McKenzie et al the authors had shown that while CITK-699A exhibits an interaction with Kif14, Kif23, and Aurora B, CitK only interacts with Kif14 (not much different than Citk699A). Whereas in this experiment in Fig 5A, CitK WT interacts with Kif14, Kif23, and Aurora B. This is obviously inconsistent. Perhaps there is a technical difference (tags), but this inconsistency must be addressed.
****

Reviewer 3: SUMMARY OF THE ADVANCE MADE IN THIS PAPER AND ITS POTENTIAL SIGNIFICANCE TO THE FIELD

SUGGESTIONS TO AUTHORS
I have read the rebuttal and looked at the relevant parts of the revised MS. I realise now that I did miss antibody quality controls, Figs 2D and F, and thank the authors for pointing that out.
Overall I am much happier with it and think the rebuttal addresses many of my initial concerns.
I would now recommend publication subject to some minor revisions:

Minor comments

Figure 1 comments
Apologies I read maltose binding protein for MBP, rather than myelin basic protein.
I still think it's important to show that the proteins were added, despite a positive phosphorylation - this can the be used to normalise/ quantitate the radiation signal. However, as bands are very clear in this example, I accept the rebuttal. N=2
1E I would still like to see labels on the figure rather than in the legend.

Figure 2:
2E -my comment was simply asking for majority mitotic stage to be noted above the lanes in the blot.
Figure S2. The authors state that they "have no evidence indicating that expression of GFP::CIT-K S440A may affect the expression" or stability of the endogenous protein.- can this be included for completeness - blot of endogenous protein.

Figure 4. I would still like to see separation of binucleates from those cells with more than 2 nuclei.

© 2026. Published by The Company of Biologists under the terms of the Creative Commons Attribution License (https://creativecommons.org/licenses/by/4.0/).

Second revision

Author response to reviewers' comments

Reviewer 2

****

The updates to my previous comments are presented following the comments from the initial review. Such comments are bracketed by *** like this one.

****

****

Many of the issues from the previous reviews were not adequately addressed.

****

1) Throughout the manuscript, including the title, abstract and main text, the authors indicate that the phosphoregulation is "coordinated",that there is "cross-talk", or there is a "cross regulatory mechanism". These terms imply that one phosphorylation event impacts the other, but there is no data provided to support that finding. Thus, these terms should be removed. A discussion of the general existence of crosstalk also appears in the introduction, which is also unnecessary.

****

In the revised version the abstract states "Here, we report the characterization of a novel cross- talk mechanism by which CDK1 and Aurora B (AURKB) kinases regulate the distribution and interactions of Citron kinase (CIT-K)."

Likewise the end of the discussion states,
"Here, we report a novel cross-regulatory mechanism among mitotic kinases important for the formation and stability of the midbody."

No evidence for cross-talk is reported or demonstrated in this manuscript.

****

We already explained in our previous response that we think that the use of the cross-talk term is appropriate because we present evidence of a cross-regulative mechanism between mitotic kinases.

2) While these studies are conducted in Hela cells which are known to require citron kinase. However, this general requirement for cytokinesis is not supported by mouse genetic analysis in which citron kinase is primarily required in the brain (PMID 11086988). This important piece of background information should be included.

****

The fact that mice lacking citron kinase are viable (and male sterile) shows that Citron kinase is not generally required for cytokinesis in all vertebrate cells. The authors reply states that "Finally, we do not investigate the role of CIT-K phosphorylation in brain development and therefore it would not be relevant to discuss this topic in our manuscript." yet the manuscript states that "Finally, some midbody proteins have been linked to brain development and microcephaly (Siskos et al., 2021)." Why don't the authors want to provide readers with the full biological context?
Likewise, Figure 1D only shows the presence of these sites in vertebrates. Are these sites conserved in invertebrates?

****

In our previous reply to this comment, we pointed out that CIT-K is required for cytokinesis in a variety of human cell lines. We mention in the Introduction that CIT-K and other midbody proteins are particularly important for brain development in rodents and humans, and think that it is not necessary to expand this part further. This is a research article, not a review.

As for the conservation of these two serine residues in invertebrates, the identity

© 2026. Published by The Company of Biologists under the terms of the Creative Commons Attribution License (https://creativecommons.org/licenses/by/4.0/).

between the human and Drosophila orthologs is too low to unequivocally identify the corresponding residues (PMID: 15240570).

3) The following contradictory sentences appear on pages 6 and 7 respectively.
"These results indicated that the antibodies specifically recognize CIT-K phosphorylated at S440 and S699."
"Notably, the pS440 signal to the midbody core and arms did not colocalize with CIT-K::GFP and did not always disappear after CIT-K RNAi (Fig. 3), suggesting that it might not be specific for CIT-K. However, we cannot exclude that this signal may represent a relatively small, but particularly stable population of CIT-K pS440."
"Unfortunately, the CIT-K pS669 antibody failed to detect any specific signal that was reduced or eliminated after CIT-K RNAi, indicating that this phospho-antibody is not suitable for immunofluorescence experiments (Supplementary Fig. S1)."

Given that the CIT-k siRNA fully depletes the CIT-k signal at the bridge, it is highly unlikely that the residual signal with the phospho-antibody is detecting a stable population. Indeed, while these antibodies have utility for western blotting where reactive species can be size separated, they are not useful for IMF either due to non-specificity (fig 3) or non reactivity (fig S1). Thus these figures are not informative and should not be included as they do not provide any new, reliable information.

****
While it is reasonable to conclude that the pS440 shown in Figure 3 "early cytokinesis" does represent phosphorylated CitK as it colocalizes with the tagged protein and is sensitive depletion of CitK by RNAi it is incorrect to conclude that the signal observed in "mid" and "late" cytokinesis represents pS440 CitK as (1) this signal does not colocalize with GFP-CITK and (2) is not subject to depletion by CitK RNAi. The argument for differential detection are unconvincing, the signals in early cytokinesis are comparable, why would this change later during cytokinesis? Bottom line, the phospho S440 antibody fails both tests for specificity in late cytokinesis, so it is not clear why the authors would want to include results that are entirely unconvincing.
****

We already addressed this comment in our previous reply. We do not conclude that the signals observed in mid and late cytokinesis cells are specific for CIT-K. We explicitly say that these signals might not be specific (page 7): "*The pS440 signals in metaphase, anaphase and early cytokinesis cells colocalize with GFP::CIT-K and are reduced or absent after CIT-K RNAi, indicating that they specifically detect pS440 CIT-K (Fig. 3). By contrast, the pS440 signals to the midbody core and arms in mid and late cytokinesis cells did not colocalize with CIT-K::GFP and did not always disappear after CIT-K RNAi (Fig. 3), **suggesting that they might not be specific for CIT-K***". There is no valid reason to exclude these relevant results.
****
3B) The origin of graph in fig 2F should be set to 0, not 0.4 (as in Figure 2C)
****
The origin of the y-axis in the graph in Figure 2F was set to 0.4 because there are no data values below this point. We have modified the graph as requested by the Reviewer.

4) Functional analysis 1 - figure 4
The strongest effect on cytokinesis is observed with CIT-K699A. However, this is not dominant (no effect in the absence of siRNA), and yet the degree of multinucleation is far higher than depletion of CitK itself.

****
The point I was making is that the inhibitory effect of CIT-K 699A is not observed in the presence of the WT protein, indicating that this is not acting as a dominant negative. Furthermore, the effect of this allele is greater than the loss of CIT-K itself, suggesting it is not a loss of function phenotype, together that indicates that CIT-K is a gain of function interfering allele. The lack of dominance could be that in the presence of the WT, a large fraction of this protein dimerizes with the WT and AurB phosphorylation of either subunit suffices to prevent its from acting dominantly. This is consistent with Fig 7f of McKenzie et al.

© 2026. Published by The Company of Biologists under the terms of the Creative Commons Attribution License (https://creativecommons.org/licenses/by/4.0/).

In any case, given that both S440D and S699D largely rescue multinucleation that dynamic phosphorylation at these sites are not essential. This key finding should be clearly stated.
****

We addressed this point in our previous reply and are sincerely confused by these additional comments. We think that it is important to clarify a few points:

a) HeLa T-REx cells are homozygous for two wild type *CIT* alleles. Transgenes are inserted in a different genomic location using the FLP-FRT recombination system. Therefore, they cannot be considered *CIT* alleles.
b) We have no evidence indicating that the S699A CIT-K mutant interferes with wild type CIT-K. Consistent with this, the phenotypes caused by CIT-K S699A expression can only be observed after depletion of endogenous CIT-K. These results, along with those previously reported in McKenzie et al. (2016), clearly indicate that phosphorylation at this site is important for cytokinesis and proper CIT-K localization.
c) Expression of S440D CIT-K affects midbody assembly and stability, with an impact on abscission and inheritance of midbody remnants (Fig. 4). Therefore the Reviewer's conclusion that phosphorylation at this site is not important is incorrect.

5) Functional analysis 2 - figure 5
This figure is fundamentally flawed. In this experiment, a key control experiment is repeated twice with diametrically different results. In fig 5 A and C, CitK is depleted by RNAi and reconstituted with wild-type CitK. In A, GFP CITK WT efficiently pulls down Kif14, MKLP1 and AURKB, whereas in C only a small amount of Kif14 is recovered, and MKLP1 and AURKB are undetectable. This lack of reproducibility calls into question that results with the phosphomutants. Given that S440D variant generates large, odd looking midbodies, it is possible that its negligible recovery in 5A is due to it being pelleted after lysis.
****

This analysis remains fundamentally flawed. The lack of consistency between Fig 5A and 5C in the WT lanes is not adequately explained by differential exposures. None of the exposures shown in the supplement support this notion.
We explained in our previous reply that CIT-K interacts with KIF14, MKLP1 and Aurora B in all our experiments and that the difference in the intensity of the MKLP1 and Aurora B signals between Fig. 5C and Fig. 5A is because the exposure time of the Western blot in Fig 5C was shorter. This is supported by quantitative analysis (Fig. 5D) and consistent with our previously published experiments using a different cell line expressing the S699A mutant (McKenzie et al., 2016).
We do not show an overexposure of the Western blot in Fig. 5C because, as described in Materials and Methods (page 15): "*chemiluminescent signals were acquired below saturation levels using a G:BOX Chemi XRQ (Syngene)*".

Note the authors should clarify the images in this supplemental figure. It is apparent, but not-stated that the filters were cut up, probed with different antibodies and reassembled. In the reassembled figure it is not transparent which part of the filters were probed with which antibody
Yes, the membranes of these Western blots were divided in strips to be probed with different antibodies generated in the same host species. This method was designed to correctly quantify the amount of prey proteins because membrane stripping and the use of membranes from different Western blots can create incorrect and misleading results. We have now included this information in the Material and Methods and in the legend of Fig. S7. Please note that the labelling clearly indicate that the order of the cropped images is identical in Figure 5 and Figure S7. The cropped images are clearly marked by red rectangles in Fig. S7.

Secondly, the failure to recover GFP-CITK-S440D in the purifications renders the assessment of its interacting proteins moot. It is a failed experiment. Why publish an experiment that failed due to technical reasons?

© 2026. Published by The Company of Biologists under the terms of the Creative Commons Attribution License (https://creativecommons.org/licenses/by/4.0/).

This comment is incorrect; GFP::CIT-K S440D is present in the purification shown in Fig. 5A.

Third, in Fig 7fe of McKenzie et al the authors had shown that while CITK-699A exhibits an interaction with Kif14, Kif23, and Aurora B, CitK only interacts with Kif14 (not much different than Citk699A). Whereas in this experiment in Fig 5A, CitK WT interacts with Kif14, Kif23, and Aurora B. This is obviously inconsistent. Perhaps there is a technical difference (tags), but this inconsistency must be addressed.
****

This comment is not very clear. The results shown in Fig. 5C in this manuscript and in Fig. 7e of McKenzie et al. (2016) are very similar, as shown in the combined figure below. The data shown in Fig. 5A are from cell lines expressing S440 phospho mutants and therefore cannot be compared with Fig. 7e of McKenzie et al. (2016).

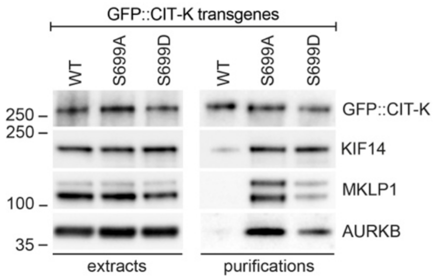

Fig. 5C (this study)

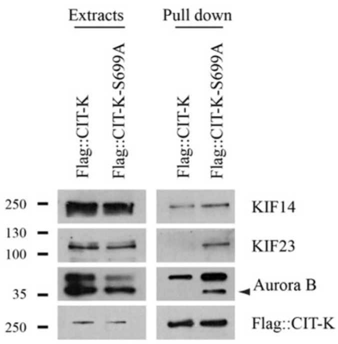

Fig. 7e (McKenzie et al., 2016))

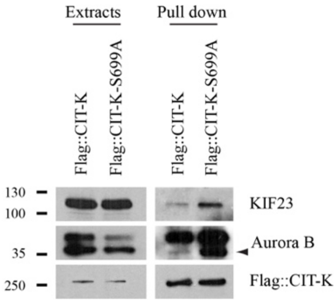

Fig. S5 (McKenzie et al., 2016)

[NOTE: Fig. 7e and Fig. S5 from McKenzie et al., 2016 are reproduced under the terms of a CC BY 4.0 licence.]

Reviewer 3

I have read the rebuttal and looked at the relevant parts of the revised MS. I realise now that I did miss antibody quality controls, Figs 2D and F, and thank the authors for pointing that out. Overall I am much happier with it and think the rebuttal addresses many of my initial concerns. I would now recommend publication subject to some minor revisions:
We are pleased that the Reviewer recommends publication subject to minor revisions. Our responses are listed below.

Minor comments
Figure 1 comments
Apologies I read maltose binding protein for MBP, rather than myelin basic protein. I still think it's important to show that the proteins were added, despite a positive phosphorylation - this can the be used to normalise/ quantitate the radiation signal. However, as bands are very clear in this example, I accept the rebuttal. N=2 1E I would still like to see labels on the figure rather than in the legend.
We have labelled the model of the CIT-K structure in Fig. 1E as requested by the Reviewer.

Figure 2:
2E -my comment was simply asking for majority mitotic stage to be noted above the lanes in the blot.
We do not have this information because cells were collected and directly processed for protein extraction. No immunostaining was carried out. Mitotic exit can be monitored by Cyclin B degradation. In our experience, HeLa cells are in ana/telophase 90 minutes after nocodazole release.

Figure S2. The authors state that they "have no evidence indicating that expression of GFP::CIT-K S440A may affect the expression" or stability of the endogenous protein.- can this be included for completeness - blot of endogenous protein.

© 2026. Published by The Company of Biologists under the terms of the Creative Commons Attribution License (https://creativecommons.org/licenses/by/4.0/).

The endogenous CIT-K protein is the faster migrating band in every panel in Fig. S3 (formerly S2).

Figure 4. I would still like to see separation of binucleates from those cells with more than 2 nuclei.
We included binucleate cells in the total count of multinucleate cells. However, the vast majority of multinucleate cells were binucleate.

Third decision letter

MS ID#: jcs.264556R3

MS Title: Regulation of Citron kinase by CDK1 and Aurora B regulates midbody formation and stability

Authors: Luisa Capalbo; Ella Halcrow; Zuni Bassi; Pier Paolo D'Avino

Article Type: Research Article

Dear Paolo,

Thank you for considering the comments raised by reviewer 2 and submitting a revised version of you manuscript.

I am happy to tell you that your manuscript has been accepted for publication in Journal of Cell Science, pending standard publication integrity checks.

© 2026. Published by The Company of Biologists under the terms of the Creative Commons Attribution License (https://creativecommons.org/licenses/by/4.0/).
